# Design of a multi-epitope protein as a subunit vaccine against lumpy skin disease using an immunoinformatics approach

**DOI:** 10.1038/s41598-022-23272-z

**Published:** 2022-11-12

**Authors:** Prajna Parimita Kar, Prasanna Babu Araveti, Akshay Kuriakose, Anand Srivastava

**Affiliations:** 1 National Institute of Animal Biotechnology (NIAB), Gachibowli, Gopanpalli, Hyderabad, 500 032 Telangana India; 2 Regional Centre for Biotechnology (RCB), Faridabad, 121 001 Haryana India

**Keywords:** Virology, Vaccines

## Abstract

Lumpy skin disease (LSD) is a transboundary viral disease of cattle that causes substantial economic loss globally. There is no specific treatment and subunit vaccine for this disease to date. Reports of the global spread of this disease are worrisome. We designed a multi-epitope protein using an immunoinformatics approach in this study. We analyzed the proteome of LSDV and found 32 structural/surface proteins. Four of these 32 proteins were predicted as antigenic and non-homologous to bovine and highly conserved in 26 LSDV isolates. The predicted B-cell epitopes and CTL epitopes were stitched together with the help of an AAY linker leading to the formation of a multi-epitope protein. The in silico study revealed that the modeled subunit vaccine candidate and TLR4 receptor interact with high affinity. This interaction was also found to be stable using a molecular dynamics simulation study. Our study demonstrates a leap towards developing a subunit vaccine against LSD.

## Introduction

Lumpy skin disease (LSD) is a transboundary viral disease of cattle caused by the lumpy skin disease virus (LSDV). LSDV is an enveloped virus belonging to the family *Poxviridae* and genus *Capripoxvirus.* The LSDV is a double-stranded DNA virus. The genome size of LSDV is 151 kb. The genome consists of 156 putative genes^1^ of which 146 genes are conserved that encode proteins involved in transcription, mRNA biogenesis, virion structure, and assembly^1^. The genes of LSDV are akin to the genes of other viruses of the *Capripoxvirus* genus, e.g., sheeppox virus (SPPV) and goatpox virus (GTPV)^2^. LSDV has a limited host range. It only causes infection in cattle (*Bos indicus* and *Bos taurus*) and buffaloes (*Bubalus bubalis*)^3^. LSD leads to high morbidity and low mortality in infected animals. The high morbidity caused by LSD significantly lowers the productivity of animals^4^. The clinical signs of the disease include fever, enlarged lymph nodes, drop in milk production, anorexia, abortion, infertility, and characteristic nodules on the skin^4^. The clinical symptoms of LSD was first described in Zambia in 1929^5^. The present geographical distribution of this disease is spread over in Austalian, African, European and Asian countries^6,7^. Acknowledging the economic impact of LSD, the World Organization of Animal Health (OIE) categorized LSD as a notifiable disease^8^. Also, due to the ability of this virus to spread rapidly, it is considered an agro-terrorism agent^9^.

Clinical signs such as skin nodules observed on the face, neck, udder, limbs etc., are used to diagnose LSD. The laboratory diagnosis of LSDV is primarily based on qPCR approaches that are mostly genus-specific^10–12^. Assays are also designed to differentiate between the wild-type and the vaccine strains^13,14^. Serology is based on neutralization tests but cannot discriminate antibodies (raised by infection/immunization) or virus species (SPPV, GTPV, or LSDV)^15–17^.

No chemotherapeutic drugs are available for the treatment of LSD and there are no subunit vaccines for the control of LSD. Current control of this disease is possible through vaccination with the live attenuated SPPV vaccine but is restricted to those countries where there is an overlap between SPPV, GTPV and LSDV infection^4^. Also, live attenuated LSDV vaccine strains like Neethling have been developed^4^. However, live attenuated/inactivated vaccines are widely used and considered effective for veterinary usage, but they have limitations. Furthermore, the reports of recombination of LSDV vaccine strains and field strains^18,19^ are worrisome. Also, there are chances of revival of live attenuated virus in the immunocompromised animals. These issues with live attenuated virus strategies for LSDV compelled us to look for an alternative like a subunit vaccine proactively. The subunit/multi-epitope-based vaccines are generally considered safer and cost-effective compared to the live attenuated vaccines^20^. Hence development of a subunit/multi-epitope vaccine for LSD is highly desirable^21^.

Immunoinformatics integrates computational and molecular immunological tools for identifying target antigens for vaccine development^22,23^. The exploitation of the immunoinformatics approach before experimental analysis is advantageous as it saves both time and money in addition to the experimental efforts. Furthermore, it can also narrow down a vast number of targets to a few potential molecules. Numerous studies have shown that multi-epitope vaccines could effectively elucidate protective immunity against influenza A, hepatitis B, and hepatitis C viruses^24–26^. Researchers have designed multi-epitope vaccine candidates for zoonotic viruses such as Nipah Virus, Crimean Congo Hemorrhagic Fever virus, Marburg virus, Ebola virus, and Monkeypox virus^27–31^. Also, few studies have shown that subunit vaccines against foot and mouth disease virus can be effective for veterinary applications^32–34^.

We used an immunoinformatics-driven approach in this study to design a subunit vaccine candidate against LSDV that could elicit a protective humoral and cellular immune response. Here, we designed a novel multi-epitope protein from LSDV in such a way that it displays various antigenic epitopes from LSDV.

## Results

### LSDV031, LSDV090, LSDV103, and LSDV109 are potential antigenic proteins of LSDV

**Figure 1:**
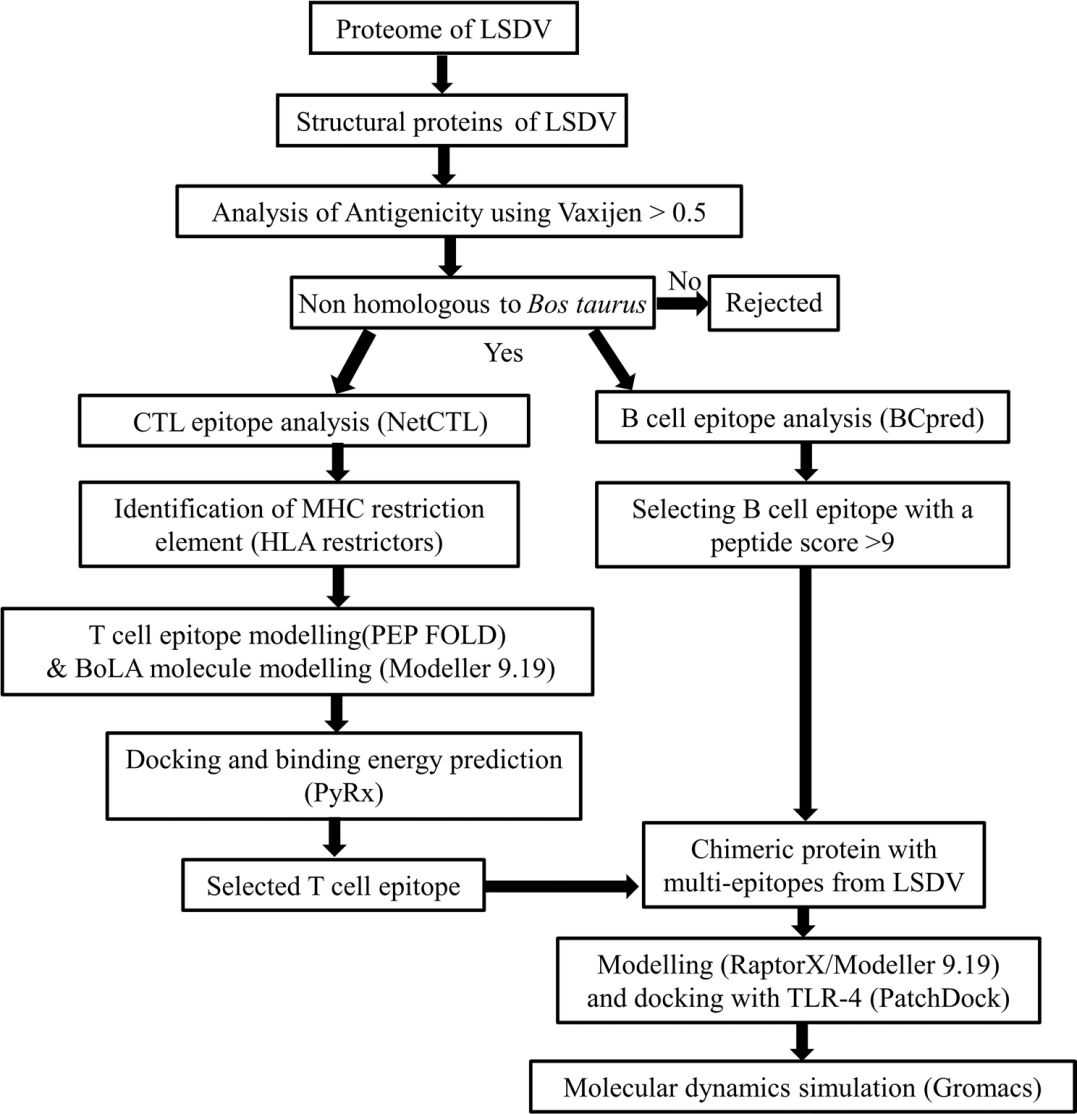
Systemic representation of designing a multi-epitope protein.

A systematic workflow was designed using the immunoinformatics approach to identify the potential vaccine candidates from the proteome of the LSDV (Fig. 1). We subsequently designed a multi-epitope protein that would possess both B-cell and CTL epitopes for eliciting an effective antibody response and cell-mediated immune response against LSDV.

We screened 32 structural/surface proteins from LSDV^1^ (Supplementary Sheet S1). All these 32 structural/surface proteins were submitted to the Vaxijen server to predict the antigenicity of these proteins. Only 5 of these 32 proteins were predicted to be potential antigens (Table 1). The prediction accuracy by the Vaxijen server is estimated to be 70–89%^35^. The potential antigenic proteins were further analyzed for their non-homology with the host (Bovine). We found that a protein with the gene-ID LSDV123 was homologous to the bovine c type lectin domain family 7 member A isoform X2 (ID-XP_005207118.1), whereas the other 4 proteins with gene-IDs as LSDV031, LSDV090, LSDV103, and LSDV109 were non-homologous to bovine (Table 1). These 4 proteins were selected to construct a multi-epitope protein as a subunit vaccine candidate against LSDV.

**Table 1:** List of antigenic proteins LSDV and proteins non-homologous to bovine.

S. No	Gene ID	Antigenic	Score	Non-homologous at E = 0.005
1	LSDV031	Antigenic	0.52	Non-homologous
2	LSDV090	Antigenic	0.51	Non-homologous
3	LSDV103	Antigenic	0.55	Non-homologous
4	LSDV109	Antigenic	0.55	Non-homologous
5	LSDV123	Antigenic	0.59	Homologous

All the 4 target sequences were subjected to BLAST search against the proteome of 27 different strains of LSDV and we found that all 4 proteins, namely LSDV090, LSDV031, LSDV103 and LSDV109, were present in the twenty-seven species (Supplementary File 1). The Clustal Omega analysis suggested that LSDV031 has 100% identity in all the isolates, while only one indel was identified for LSDV090 and LSDV109 at positions T277S and F181V, respectively. For LSDV103 we found indels at T49N, K71T, P72T, and S89G. This analysis suggests that all the selected proteins are highly conserved in LSDV.

### The selected antigenic proteins of LSDV possess B-cell epitopes

The selected 4 antigenic proteins were further analyzed for the prediction of linear B-cell epitopes with a twenty amino acid long peptide. The peptide with a score greater than 9 on a scale of 10 was selected as a potential B-cell epitope to minimize the chance of selecting the false-positive epitopes. With the transmembrane analysis using TMHMM, we found that LSDV109 contains 3 transmembrane domains and LSDV031 is an intracellular protein, whereas the other 2 proteins, namely LSDV090 and LSDV103, are extracellular proteins. LSDV031 being an intracellular protein was removed from the B-cell epitope analysis. We found that all 3 proteins, namely LSDV090, LSDV103, and LSDV109, contain 15 B-cell epitopes. However, the B-cell epitope “FTREEEEAFLPKEHHEEGEE” from LSDV109 was predicted in the transmembrane region, so it was not considered as a B-cell epitope (Table 2). Interestingly, LSDV090 protein alone contained 9 of these 14 B-cell epitopes, suggesting that this protein could be tested as a potential subunit vaccine candidate for LSDV.

**Table 2:** Predicted B-cell epitopes from the selected proteins of LSDV.

S. No	Gene ID	Position	Epitope	Score
1	LSDV090	292	LEDLVVVSKEPPNYFPESAE	0.989
		38	TLNGVSSTGSCTQNVVSTFE	0.985
		400	WSCQRNVYNGDNRSESSKNK	0.985
		250	NSVTSFSVYVKPYYGNTDNK	0.976
		509	FSSFDPNNPIYYVSKQIVLV	0.953
		93	KSIQHVSISCNNGTIWESSG	0.925
		352	TRKNSITYNISKKFSTITGS	0.915
		483	PHTFFKPTTIVSNTARGKDK	0.912
		118	SCKNNETALNNSGFCHELNS	0.908
2	LSDV103	39	QKSNLTPEDNTTNNTDENEV	0.999
		71	KPNKKSKSCSNKQTTSRSSN	0.985
		117	QAVTNGGKIVYGTMKEGKLE	0.965
3	LSDV109	108	LSIQKFSGGVGNNKQIIMSI	0.925
		175	QACYKNFKGGNKYREKPSFY	0.905

### The selected antigenic proteins of LSDV possess cytotoxic T lymphocytes (CTL) epitopes specific to bovine MHC-I restriction elements

Four antigen proteins that were non-homologous to bovine were further analyzed for the presence of cytotoxic T-lymphocyte (CTL) epitopes using NetCTL 1.2 server against all the 12 supertypes. We selected the threshold of 0.98 for better sensitivity and specificity in predicting the CTL epitopes. Seventy-seven CTL epitopes were identified from the NetCTL server. The CTLs recognize antigens in the context of MHC-I. Hence, estimating the binding affinity of the CTL epitope and bovine MHC-I molecule is essential. All the 77 CTL epitopes were further analyzed for their binding affinity with a total of 77 bovine MHC-I molecules of *Bos taurus* available in the HLArestrictor server. Three proteins, namely LSDV031, LSDV090, and LSDV109, were found to possess 17 CTL epitopes with a strong binding affinity (IC_50_ value < 50 and percentile rank below 0.5) only with 22 different bovine MHC-I molecules (Supplementary sheet S2). Five of these 17 CTL epitopes showed a strong binding affinity with BoLA-1*00902, 5 showed a strong binding affinity with BoLA-3*05101, and 4 showed a strong binding affinity with BoLA-6*01301. Three peptides, YYANTPFYI, SERDFICVF, and KISKHHSLK showed a strong binding affinity with BoLA-5*00301, BoLA-6*01402, and BoLA-2*01201, respectively. Thus, we chose 6 MHC-I molecules, namely BoLA-1*00902, BoLA-3*05101, BoLA-6*01301, BoLA-5*00301, BoLA-6*01402, and BoLA-2*01201, out of 22 MHC-I for further analysis as these were sufficient to bind to all the selected CTL epitopes.

### The selected CTL epitopes and bovine MHC-I allele interact strongly in silico

The crystal structures for the selected bovine MHC-I, namely BoLA-1*00902, BoLA-3*05101, BoLA-6*01301, BoLA-5*00301, BoLA-6*01402, and BoLA-2*01201 proteins, were unavailable. Hence a homology model-based approach was carried out to build a reliable model structure for these selected proteins using Modeller 9.19. Only the extracellular region of these proteins was considered for modeling (Supplementary Sheet S3). The BoLA-1*00902, BoLA-3*05101, BoLA-5*00301, and BoLA-2*01201 showed 74.19%, 75.44%, 71.64%, and 78.30% identity, respectively with the structure of the F chain of the human MHC-I peptide-loading complex (PDB Id: 6ENY 'F'). The root mean square deviation (RMSD) values for BoLA-1*00902, BoLA-3*05101, BoLA-5*00301, and BoLA-2*01201 were found to be 1.116 Å, 1.614 Å, 0.976 Å, and 0.702 Å, respectively after refinement using ModRefiner.

Similarly, BoLA-6*01301 and BoLA-6*01402 showed 100% and 92.39% identity with a query coverage of 77% with cattle MHC-I N*01301 (PDB Id: 2XFX ‘A’). After refinement, the RMSD value of the modeled BoLA-6*01301 and BoLA-6*01402 were predicted to be 1.084 Å and 0.687 Å, respectively. The Ramachandran plot of all the modeled bovine MHC-1 suggested that the predicted models were stable and reliable (Table 3).

**Table 3:** Prediction of stability of modeled protein structure by Ramachandran plot.

Protein name	Core region (%)	Allowed region (%)	Generously allowed region (%)	Disallowed region (%)
BoLA-1*00902	93.1	5.8	0.4	0.8
BoLA-3*05101	94.1	5.1	0	0.8
BoLA-6*01301	93.5	6.2	0.4	0
BoLA-5*00301	94.5	4.7	0	0.8
BoLA-6*01402	94.2	5.4	0.4	0
BoLA-2*01201	92.7	5.0	0	2.3
Multi-epitope protein	80.3	16.3	1.9	1.5
Bovine TLR4	80.9	16.0	2.4	0.6
Bovine TLR2	81.2	15.5	2.1	1.1

The three-dimensional structures for all the strong binder CTL epitopes were predicted by PEP-FOLD server, and further analyzed by docking studies with their respective MHC-I molecules. The binding energy was recalculated from the docking studies. The CTL epitope ‘SQYYANTPF’ was found to bind with BoLA-1*00902 with a highest binding score of − 8.9 kcal/mol, while the CTL epitope ‘SEINSVTSF’ was found to bind with the BoLA-1*00902 with a lowest binding score of − 5.8 kcal/mol (Table 4).

**Table 4:** Binding energy of modeled bovine MHC-I with predicted CTL epitopes.

Gene ID	Peptide sequence	Binding energy (kcal/mol)
BoLA-1*00902	SQYYANTPF	− 8.9
	KESATIYVY	− 7
	FIYVTELSF	− 8
	SEINSVTSF	− 5.8
	FRHISSTAY	− 7.8
BoLA-3*05101	QYYANTPFY	− 6.6
	FGYVTYVGY	− 8.6
	YYHSNILVF	− 7.2
	SFSVYVKPY	− 6.8
	SYYNMFSDF	− 7.9
BoLA-6*01301	LQAKNRSVM	− 6.6
	IMIAVASAL	− 7.3
	YKISKHHSL	− 7.6
	8LMRSDIRAL	− 8.5
BoLA-5*00301	YYANTPFYI	− 8.6
BoLA-6*01402	SERDFICVF	− 7.1
BoLA-2*01201	KISKHHSLK	− 8.6

### Engineered multi-epitope protein is antigenic and non-allergenic

**Figure 2:**
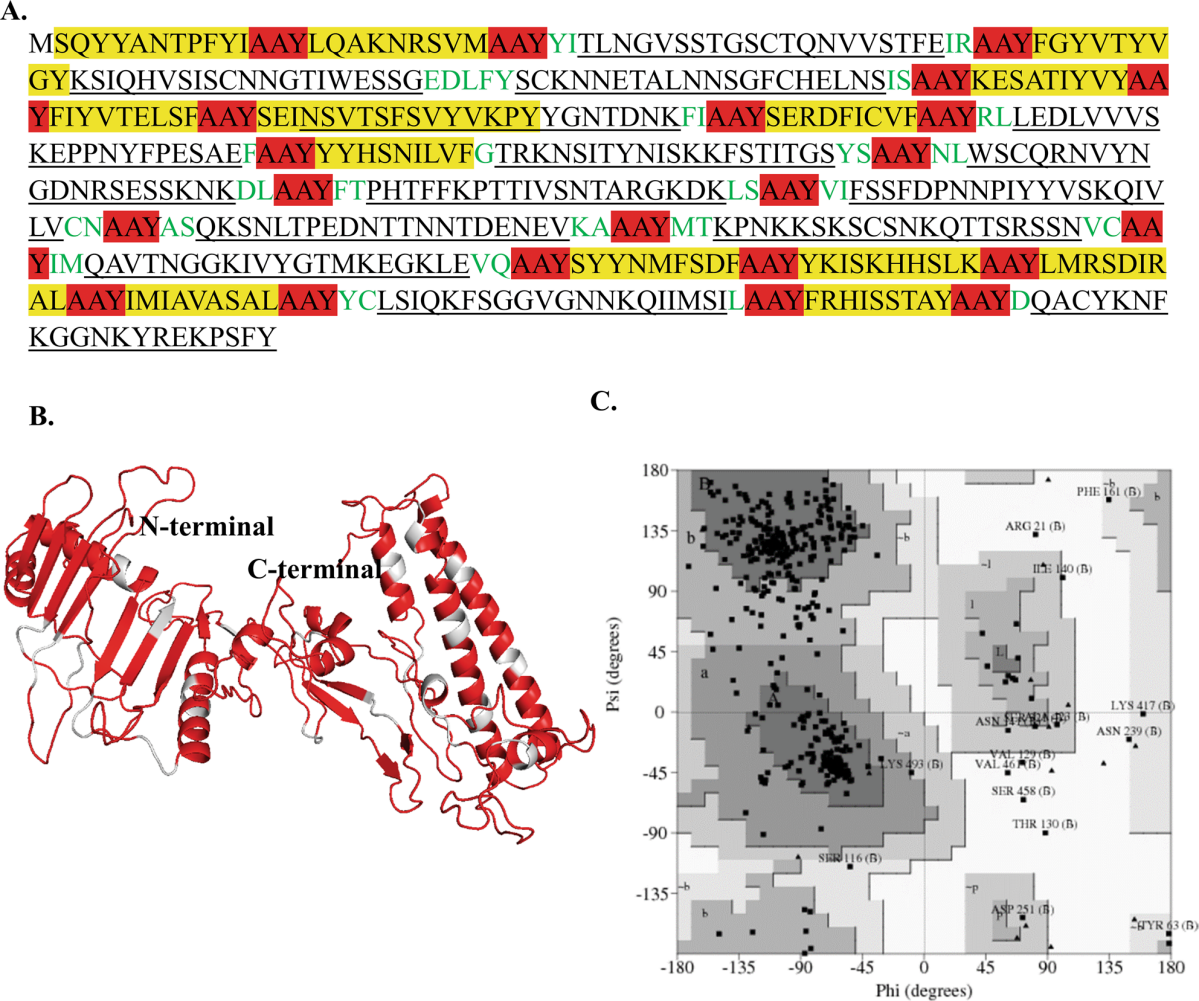
Modeled structure of multi-epitope protein against LSDV. (**A**) Protein sequence of multi-epitope protein. Sequence in underline: B-cell epitope; yellow color: CTL epitope; green color: extra sequences for the stability of multi-epitope protein; Highlight red color: AAY linker, (**B**) Three-dimensional model of multi-epitope protein obtained by modeling and refinement by RaptorX. The gray color represents AAY linker, (**C**) Ramachandran plot analysis of the multi-epitope protein showing favored (80.3%), allowed (16.3%), generously allowed (1.9%), and disallowed regions (1.5%).

Before joining the selected peptides, the toxicity analysis was performed for all the selected 17 peptides using toxinpred^36^. The toxicity analysis suggests that none of the peptides would be toxic in nature (Supplementary sheet S4). Thus, we engineered a multi-epitope protein against LSDV by joining all the predicted CTL epitopes and B-cell epitopes with an AAY linker (Fig. 2A). We selected the regions around the B-cell epitopes from these proteins to prevent loss of B-cell epitope because of the presence of an AAY linker in the multi-epitope protein. This led to the design of a multi-epitope protein with a total length of 508 amino acids (Fig. 2A). The designed multi-epitope protein was evaluated for presence of any allergenic representative peptide in the new protein by AlgPred. AlgPred BLAST search on allergen representative peptides (ARPs) showed that the designed multi-epitope protein was non-allergenic. The molecular weight and theoretical pI of this multi-epitope protein were calculated as 56.8 kDa and 9.02, respectively. The designed multi-epitope protein was slightly basic based on the theoretical pI calculation. The instability index of 37.95 represents the stable nature of the multi-epitope protein. The designed protein was found to have an aliphatic index of 69.43, showing that this can be thermally stable at variable temperatures. Further, we found using FoldIndex server that the multi-epitope protein would fold properly since the unfold-ability index is 0.141. Also, the antigenicity score of 0.5624 shows that this multi-epitope protein would be antigenic.

### The predicted tertiary structure of the multi-epitope protein, bovine TLR4 and TLR2 is stable

RaptorX server was used for the prediction of a three-dimensional model structure of the designed multi-epitope protein (Fig. 2B). The Ramachandran plot of this protein revealed that 80.3% amino acids were located in the core region, 16.3% amino acids were located in the allowed region, 1.9% amino acids were located in the generously allowed region and 1.5% amino acids residues were found in the disallowed region (Fig. 2C, Table 3).

The crystal structure of bovine TLR4 and TLR2 are unavailable. Hence, a template for bovine TLR4 and TLR2 was obtained from its sequence for structure alignment using BLASTp search. The human TLR4 complex with MD-2 and LPS (PDB Id: 4G8A_A) was found to have 71.88% identity and 72% coverage with the bovine TLR4. Similarly, the human TLR2 (PDB Id: 6ING_A) was found to have 68.37% identity and 69% coverage with the bovine TLR2. A reliable model for bovine TLR2 and TLR4 was predicted using Modeller 9.19 (Figs. S2A and 3A). The modeled bovine TLR2 and TLR4 structures were refined with ModRefiner and validated with PROCHECK. The RMSD value obtained after refinement was found as 1.870 Å and 1.816 Å for TLR4 and TLR2, respectively. The Ramachandran plot of the modeled bovine TLR4 and TLR2 showed that these modeled structures are reliable (Table 3).

**Figure 3:**
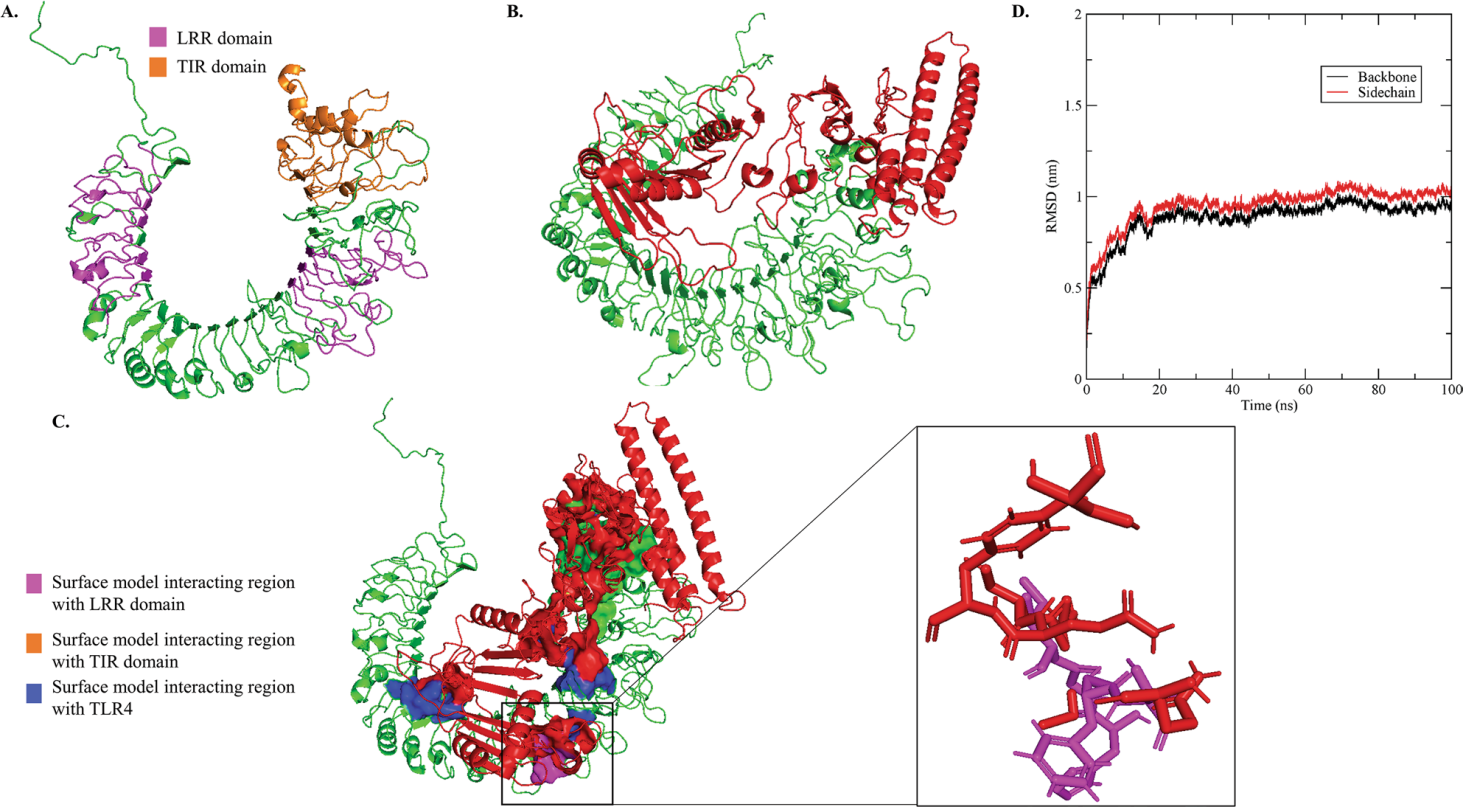
Docking studies of multi-epitope protein-based subunit vaccine candidate against LSDV and modeled bovine TLR4. (**A**) Three-dimensional model of bovine TLR4 obtained by homology modeling and refinement. LRR domain and TIR domain shown in magenta and orange color, respectively, (**B**) Docked complex of TLR4 and multi-epitope protein based subunit vaccine candidate, where green color represents the TLR4, red color represents the multi-epitope vaccine candidate, blue color and green color with surface models showed the interacting region of the multi-epitope protein and bovine TLR4, magenta with surface model showed the interacting region of LRR domain of TLR4 with multi-epitope protein, (**C**) Binding residue of the LRR domain of the TLR4 and multi-epitope protein with sticks model, (**D**) Molecular Dynamics Simulation of multi-epitope protein and bovine TLR4 for 100 ns.

### Designed multi-epitope protein against LSDV contains CTL and B-cell epitopes

The linear amino acid sequence of multi-epitope protein against LSDV was further analyzed for the presence of B-cell and CTL epitopes using BCPREDS and NetCTL, respectively. From BCPREDS analysis we found that the multi-epitope protein contains a total of 14 B-cell epitopes and all the B-cell epitopes were identical to the previously predicted epitopes. Similarly, we found from the NetCTL and HLArestrictor analysis that the multi-epitope protein against LSDV possesses a total of 30 CTL epitopes. Of these 30 CTL epitopes, 17 were previously identified as CTL cell epitopes, and 13 newly predicted CTL epitopes were identified. All the 13 newly predicted CTL epitopes were further verified for their homology with bovine, and we found none of these 13 epitopes were present in bovine (Supplementary Sheet S5). Thus, our analysis suggests that the designed multi-epitope protein would not generate autoimmunity in the bovine.

### The interaction of bovine TLR4 and designed multi-epitope protein is stable

The geometric shape complementarity score of the multi-epitope protein and bovine TLRs (TLR2 and TLR4) were calculated using the PatchDock server from the refined modeled structure of both proteins. Twenty protein–protein complexes were generated based on the protein surface, geometry and electrostatic complementarity. Top 10 of these 20 protein–protein complexes were again submitted to the FireDock to re-score the complexes according to their global binding energies. The final docking model for TLR2 and multi-epitope protein was found to be 6.5 kcal/mol (least binding energy score) this suggests that TLR2 would not interact with the multi-epitope protein (Fig. S2B). Hence, TLR2 was not considered in the subsequent analysis. The final docking model of TLR4 and multi-epitope protein was found to be − 13.71 kcal/mol (least binding energy score) and hence was chosen for further analysis. This complex was found to have the attractive van der Waals force and repulsive van der Waals force with − 11.67 kcal/mol, and 5.26 kcal/mol, respectively. We further validated the interaction of TLR4 and multi-epitope protein using ClusPro server^37^. The binding energy calculated from the PatchDock and ClusPro server were found to be − 21.6 kcal/J and − 16.7 kcal/J, respectively (Figs. 3 and S1). The results show that the multi-epitope protein binds strongly with the bovine TLR4 receptor in silico (Fig. 3B,C). The molecular dynamics simulation was carried out for 100 ns to study the stability of the TLR4-multi-epitope protein complex. The molecular dynamics simulation showed that the complex was stable in SPC water, and the root mean square deviation (RMSD) of the protein backbone and sidechain was stable after 20 ns (Fig. 3D). Further, it was found that the TLR4 and multi-epitope protein complex possesses binding free energy of − 77.03 kcal/mol using MMGBSA analysis performed at the HawkDock server. A total of 47 hydrogen bonds were formed between the TLR4 receptor and the multi-epitope vaccine protein (Fig. S3). This result evidently shows that the multi-epitope protein can form a stable complex with the bovine TLR4 in silico.

## Discussion

We used immunoinformatic tools for the prediction of the potential antigens of LSDV, which are (a) predicted to be on the surface so that it can be targeted by the host immune response, and (b) non-homologous to the host protein. We selected 32 structural/surface proteins from the LSDV proteome to identify the immunogenic proteins. All these proteins were analyzed in silico for their ability to induce an immune response. We used Vaxijen server for the prediction of potential antigens. Vaxijen is an antigen prediction server based on an alignment-free approach using autocovariance (ACC) transformation of protein sequence into uniform vectors of principal amino acid properties. Further, it is critical that a vaccine candidate should not generate autoimmunity in the host. Hence, these selected proteins were found to be non-homologous with the host proteome. The genetic diversity analysis among selected proteins showed that these proteins are conserved in various isolates of LSDV.

B-cells play an essential role in the development of humoral immunity. Although cell-mediated immunity plays a vital role in the clearance of the virus, the role of B-cells in enhancing cell-mediated immunity cannot be ignored. During viral infection, B cells function as antigen-presenting cells by presenting viral peptides to CD4 + cells through MHC-II^38^. Hence, we identified B-cell and CTL-epitopes while designing the multi-epitope protein in silico. However, due to limited resources/knowledge available to study bovine immune system, we could not perform analysis of bovine HTL epitopes. For considering HTL epitopes, we need to screen the bovine MHC-II molecules; however, to date, there are no resources/software(s) available which can be used to screen peptide sequences against the bovine MHC-II molecule.

Generally, cell-mediated immunity plays an important role in viral clearance. It has been shown that the viruses which infect dendritic cells (DC) can directly present viral antigens to CTL. However, the viruses that target other cells different from the DC cannot directly present viral antigens to CTL. In this case, cross-presentation of viral antigens by DC to activate virus-specific CTL is required^39^. Although LSDV does not infect DC, cross-presentation of viral antigen may be possible. Hence, we predicted CTL epitopes and their corresponding MHC-I molecule in the selected LSDV proteins. We observed that BoLA-1*00902, BoLA-3*05101, BoLA-6*01301, BoLA-5*00301, BoLA-6*01402, and BoLA-2*, were sufficient to bind to all the selected CTL epitopes. Hence, we confined our studies to only these MHCs. The modeled MHC-I and CTL-epitopes were docked with the help of PyRx software, and their binding energies were calculated further to validate the interaction of modeled MHC-I and CTL-epitopes.

Previously, an elegant study was conducted to identify linkers for joining multi-unit CTL epitopes^40^. This study suggested that the amino acids with aromatic (tyrosine), basic (lysine), and small aliphatic side chains (alanine) support efficient CTL recognition of both flanking epitopes. Hence, we used an AAY linker for joining the predicted CTL epitopes and B-cell epitopes. In order to understand the effect of AAY linker on the immunogenicity and stability of multi-epitope vaccine candidate, we designed the multi-epitope vaccine candidate with and without a linker. The antigenicity of the multi-epitope vaccine candidate with and without linker was found to be 0.5791 and 0.5158, respectively. This suggests that adding the linker AAY would not affect the immunogenicity of the vaccine candidate. The physiological properties of the vaccine candidate was also predicted with and without AAY linker. The instability index of the multi-epitope protein without AAY linker was found to be 41.75, suggesting that the protein would be unstable. In contrast, after adding the linker AAY the instability index of the multi-epitope protein was found to be 37.95, suggesting the protein would be stable. Further, we used the FoldIndex server to predict the unfoldability index of the multi-epitope vaccine candidate with and without a linker. We observed that the multi-epitope vaccine candidate with and without linker AAY linker would have a similar unfoldibilty index (Supplementary sheet S6). We observed that in a few cases, when 2 epitopes were joined together with the AAY linker it led to the generation of newer epitopes that were different from the viral epitopes. However, when extra amino acids continued with the epitope of the same viral protein were added to multi-epitope protein, the newer epitopes were not generated. Hence, we used the extra amino acids in the selected epitopes. As the designed multi-epitope protein is an assembly of various peptides, it is possible that the multi-epitope protein may have homology with host proteins. We performed a homology search again for the multi-epitope protein with the bovine proteome to rule out this possibility. The designed multi-epitope protein was found to be non-homologous to bovine. The calculated instability index for the primary amino acids sequence of this protein indicates that it would be a stable protein. The structure of the vaccine construct was modeled using RaptorX contact predict server. It is a threading-based modeling method that uses an ultra-deep convolutional residual neural network from a primary sequence or a multiple sequence alignment. It is possible to identify structurally similar fold(s) with the query sequence using RaptorX which can be used as a reliable template for modeling the tertiary structure of a given protein. The Ramachandran plot of the modeled tertiary structure showed that only 1.5% of the residues would fall into the disallowed region. With these results, we concluded that the multi-epitope protein is quite stable and ordered.

Toll-like receptors (TLRs) are essential in recognizing conserved molecules derived from microbes. They are characterized by an extracellular leucine-rich repeat domain and an intracellular Toll/IL-1 receptor-like (TIR) domain. Of all the TLRs, only TLR4 and TLR2 have been shown to recognize the viral proteins^41,42^. TLR4 is found on the cell surface of many cell types, such as antigen-presenting and endothelial cells. TLR4 was first identified as a sensor for bacterial components, especially LPS^43^. Later, it was found that TLR4 could respond to viral pathogens such as a respiratory syncytial virus. It was shown that TLR4 could bind to the respiratory syncytial membrane-bound fusion (F) protein leading to the induction of IL-6^44^. Recently, various other viral glycoproteins, such as Ebola virus GP^45^, VSV-G^46^, the envelope proteins of Moloney murine leukemia virus (MMLV)^47^, could bind and activate TLR4, further leading to the expression of proinflammatory cytokine expression. Thus we analyzed the potential of TLR4 receptor to interact with the modeled multi-epitope vaccine candidate*, *in silico*,* in this study. An effective CTL response against the designed multi-epitope protein would be generated only when it is presented to the CD8 + T-cells by the antigen-presenting cells. Thus, we hypothesized that bovine TLR4 present on the antigen-presenting cells would play an important role in recognizing the multi-epitope protein. The subunit vaccine candidate was found to interact with TLR4. Further, the docking and molecular dynamics simulation studies of the bovine TLR4 and the multi-epitope protein suggested that this complex would be stable. The interaction of TLR4 with subunit vaccine candidate would activate TLR4. The activation of TLR4 pathway would activate cytokines further, leading to the activation of both innate and adaptive immune systems. Thus, in this study, we designed a new multi-epitope protein of LSDV, which would likely be an effective subunit vaccine candidate against LSDV.

In conclusion, we have designed a multi-epitope protein in silico from the structural proteins of LSDV using an immunoinformatic approach. The engineered subunit vaccine protein has been found to interact with the toll-like receptor and found to be stable under in silico conditions. Although the subunit vaccines have various advantages, they may have their own limitations. One of the significant limitations is the expression and purification of the recombinant protein as a soluble protein in heterologous expression systems. It will be interesting to confirm the expression and purification of the designed multi-epitope subunit vaccine candidate. Also, the validation of predicted immunogenicity and the potential of this designed multi-epitope protein to interact with the TLR4 in vivo would require for subsequent trials in the animals. In short, our studies demonstrate a way toward developing a subunit vaccine against LSDV and put forward a new multi-epitope protein as a potential subunit vaccine candidate likely to be effective against LSDV.

## Materials and methods

### Screening of protein non-homologous to host (bovine) and antigenicity prediction

The surface/structural proteins of the lumpy skin disease virus NI-2490 isolate Neethling 2490 (Accession no. AF325528.1) reported previously^1^ were downloaded from the National Centre for Biotechnology Information (NCBI) database (https://www.ncbi.nlm.nih.gov) for the antigenicity prediction. The antigenicity of the identified surface/structural proteins was analyzed using Vaxijen server^35^. The threshold was set to 0.5 to predict the antigenicity of proteins. Further, to identify the non-homologous proteins of *Bos taurus* with LSDV surface proteins, the BLASTp server^48^ was used at a threshold of E-value at 0.005.

The proteomes of 27 isolates of LSDV were downloaded from the NCBI database. The selected proteins were searched against the proteome of these 27 isolates of LSDV and were aligned together using Clustal Omega^49^.

### B-cell epitope prediction

The selected proteins (non-homologous with *Bos taurus* and predicted to be antigenic) from the previous step were analyzed for the prediction of the B-cell epitope(s) using BCPREDS with a specificity of 75% and a peptide score of > 9^50^. BCPREDS training dataset contains 701 linear B-cell epitopes, and 701 random B-cell epitopes from Bcipep and SwissProt sequence database, respectively. The transmembrane region was predicted for all the selected proteins using TMHMM Server, v. 2.0^51^.

### Cytotoxic T lymphocytes (CTL) epitope prediction

NetCTL 1.2 server^52^ was used to predict the presence of cytotoxic T-lymphocyte (CTL) epitopes against all the 12 supertypes with a threshold of 0.98. The CTL epitopes identified from the NetCTL 1.2 were further screened for the identification of bovine MHC-I restriction element using HLArestrictor 1.1^53^ against all the 77 bovine MHC-I alleles present in the database. The strong binders were classified with a threshold of 50 for IC_50_ and 0.5 percentile rank, while the weak binders were classified with a threshold of 500 for IC_50_ and 2 percentile rank.

### Prediction of tertiary structure, model refinement, stereochemistry and conformation analysis of bovine MHC-I

The sequences of bovine MHC-I alleles, namely BoLA-1*00902, BoLA-3*05101, BoLA-6*01301, BoLA-5*00301, BoLA-6*01402 and BoLA-2*01201, were downloaded from the Immuno Polymorphism Database (IPD)^54^. The transmembrane regions for all the bovine MHC-I molecules were predicted by TMHMM Server, v. 2.0^51^. Only the extracellular region of MHC-I was used to build a reliable tertiary structure for these proteins using a homology model-based approach. The template for the homology modeling was identified by a BLASTp search against Protein Data Bank (PDB) and the three-dimensional models of selected BoLA-MHC-I proteins were built using the MODELLER 9.19 software^55^. The stereochemistry and conformation analysis of generated models for these proteins were analyzed using PROCHECK, a protein parameters analysis tool^56^ and refinement was done using ModRefiner^57^.

### Structure prediction of CTL epitopes

The PEP-FOLD server^58^ was used for the prediction of the three-dimensional structure of CTL epitopes (classified as a strong binder from the HLArestrictor 1.1). The parameter was set to generate 5 probable structures of the CTL epitopes. The structure with the lowest energies was selected as the final model for the respective epitope.

### Docking studies of selected MHC-I allele with CTL epitope

The PyRx software^59^ was used for docking studies between the strong binder pairs of MHC-I allele and CTL epitope obtained from HLA-restrictors. For BoLA-1*00902, BoLA-3*05101, BoLA-2*01201, and BoLA-5*00301, the grid was set to 106.281, 107.904, 86.508 for center X, Y, and Z coordinates, respectively, with a spacing dimension of 110.355 Å, 102.153 Å, and 113.218 Å for the X, Y, and Z coordinates, respectively. In the case of BoLA-6*01402 and BoLA-6*01301, the grid box was set to 76.564, 57.040, 23.777 for X, Y, and Z coordinates, respectively with the spacing dimension of 72.848 Å, 81.183 Å, 88.263 Å for the X, Y, and Z coordinates, respectively. The binding affinity of the CTL epitope with its receptor BoLA-MHC-I protein was measured in kcal/mol.

### Designing of the multi-epitope protein as a subunit vaccine against LSDV

A subunit vaccine candidate for LSDV was designed by fusing all the CTL and B-cell epitopes with an AAY linker. A few extra amino acids that were in continuation with the epitope of the same protein were added to maintain the integrity of the epitopes and to prevent the generation of newer epitopes after joining with the linker. The presence of homologous sequences with bovine in the designed subunit vaccine candidate was done using BLASTp. The antigenicity, allergenicity, and intrinsically unfolded regions were predicted using Vaxijen, AlgPred^60^ and FoldIndex server^61^, respectively. Also, ProtParam^62^ was used to predict the physicochemical properties, such as amino acid composition, theoretical pI, instability index etc., of the designed subunit vaccine candidate.

### Structural characterization of the subunit vaccine candidate and bovine TLR4 and TLR2

The RaptorX server^63^, is a distance-based protein folding method of structure prediction, was used for tertiary structure prediction of the designed subunit vaccine candidate. The reliable models for bovine TLR4 and TLR2 were built using homology modeling. A BLASTp search against PDB database was used to identify the best template for homology modeling for bovine TLR4 and TLR2 proteins. The three-dimensional model of bovine TLR4 and TLR2 proteins were built using MODELLER 9.19 software. The model refinement and stereochemistry analysis for the subunit vaccine candidate and above modeled bovine TLR (TLR4 and TLR2) were achieved using the ModRefiner and PROCHECK, respectively.

### Molecular docking and simulation of bovine TLR4 and TLR2 receptors with subunit vaccine candidate

The protein–protein docking server, PatchDock^64^, was used to study the interaction between the bovine TLRs (TLR4 and TLR2) and the subunit vaccine candidate. The top ten models were again submitted to FireDock server^65^ for sorting them according to their global binding energies. To further validate the interaction of TLR4 and multi-epitope protein, we used ClusPro server^37^. Further, molecular dynamics (MD) simulation study was carried out for the docked complex of the TLR4 and subunit vaccine candidate for 100 ns with a time interval of 2 fs using Gromacs software to determine the stability of the protein–protein complex. The simulation was done using Optimized Potentials for Liquid Simulations (OPLS) force filled parameters. The receptor-multi-epitope protein complex was solvated with SPCE water molecules. The isothermal-isobaric pressure (NPT) ensemble was set at 300 K (temperature) and 1 atm pressure. The root mean square deviation (RMSD) was calculated to examine the standard deviation of the backbone and sidechain of the protein. Further, to calculate the binding free energy of the complex MMGBSA analysis performed at the HawkDock server^66^.

## Supplementary Information

Supplementary Information 1.

Supplementary Information 2.

Supplementary Information 3.

## Data Availability

All data generated or analyzed during this study are included in this published article [and its supplementary information files].
