# Supplementary material for: Design of a multi-epitope protein as a subunit vaccine against lumpy skin disease using an immunoinformatics approach
Source: Sci Rep. 2022 Nov 12;12:19411. doi: 10.1038/s41598-022-23272-z (PMC9653426; doi:10.1038/s41598-022-23272-z)
Supplement: Supplementary file 1 — Supplementary Information 1. [file 41598_2022_23272_MOESM1_ESM.docx]

**Figure S1:** **Docked complex of TLR4 and multi-epitope protein based subunit vaccine candidate using ClusPro.** The green color represents the bovine TLR4 and red color represents the multi-epitope based vaccine protein. The interacting residue of the bovine TLR4 and multi-epitope based vaccine protein is shown in the surface model.


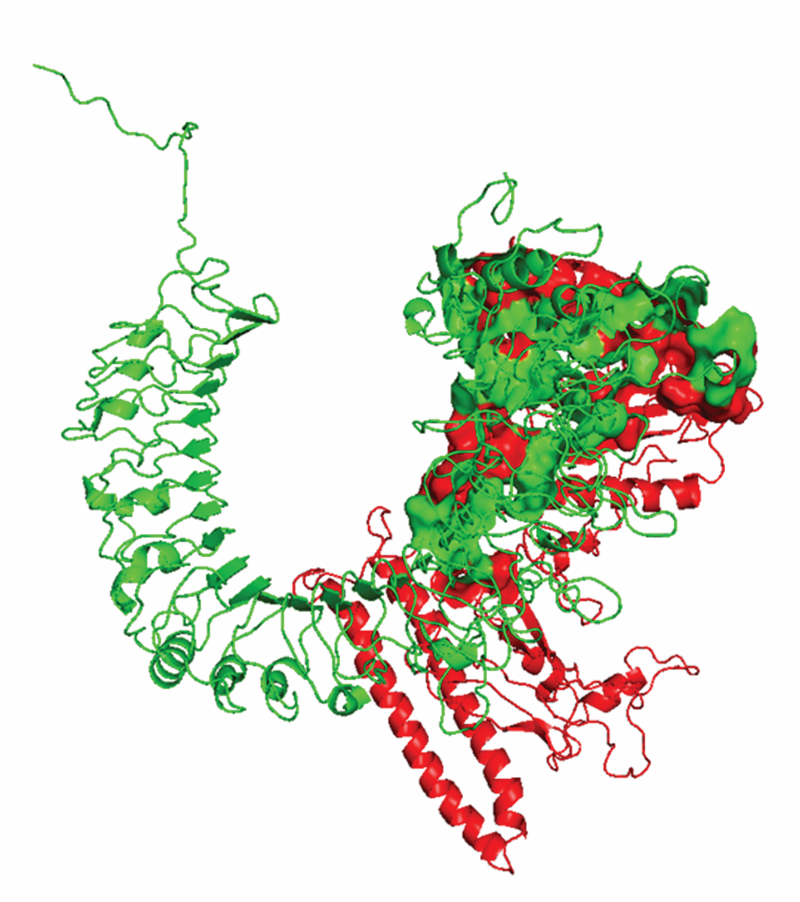


**Figure S2: Docking studies of multi-epitope protein based subunit vaccine candidate against LSDV and modeled bovine TLR2. (A)** Three dimensional model of bovine TLR2 obtained by homologous modeling and refinement with LRR with magenta color and TIR domain with orange color, (B) Docked complex of TLR2 and multi-epitope protein based subunit vaccine candidate, where green color represents the TLR2, red color represents the multi-epitope vaccine candidate. The global binding energy calculated is 6.5kcal/mol.


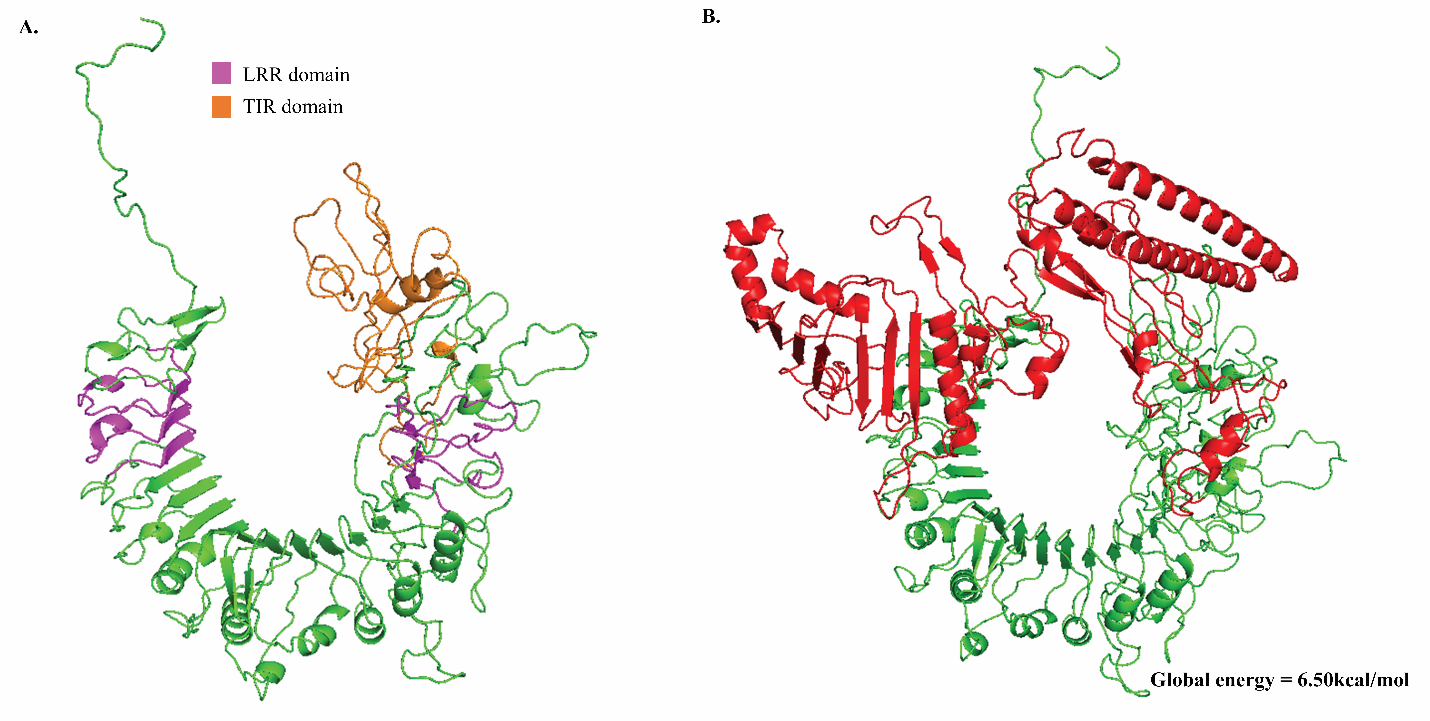


**
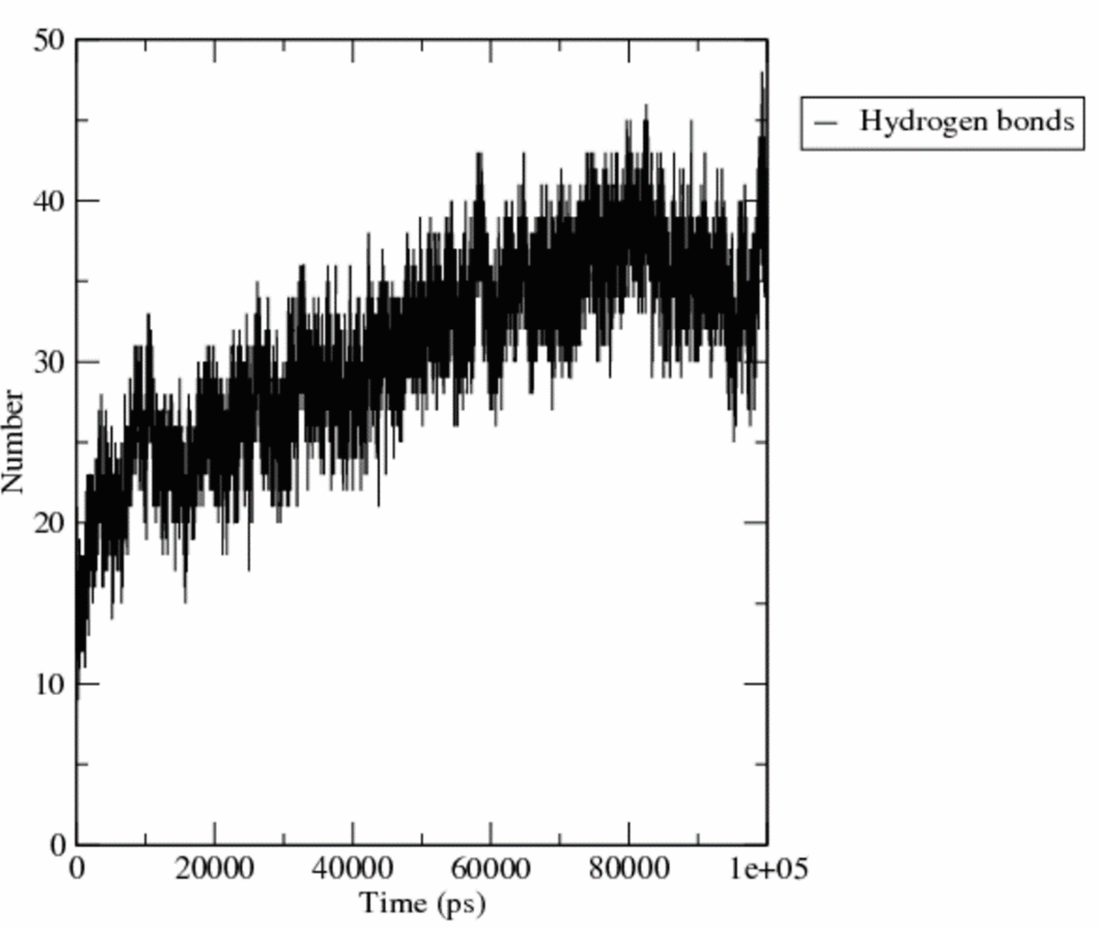
Figure S3: Number of Hydrogens bonds between TLR4 and multi epitope subunit vaccine candidate over time (100ns).**
