# Supplementary material for: Design of a multi-epitope protein as a subunit vaccine against lumpy skin disease using an immunoinformatics approach
Source: Sci Rep. 2022 Nov 12;12:19411. doi: 10.1038/s41598-022-23272-z (PMC9653426; doi:10.1038/s41598-022-23272-z)
Supplement: Supplementary file 2 — Supplementary Information 2. [file 41598_2022_23272_MOESM2_ESM.docx]

**Supplementary File 1: A.** NCBI link for LSDV isolate used in this study**.**

**1. AF325528.1:** [**https://www.ncbi.nlm.nih.gov/nuccore/AF325528.1**](https://www.ncbi.nlm.nih.gov/nuccore/AF325528.1)

**2. AF409137.1:** [**https://www.ncbi.nlm.nih.gov/nuccore/AF409137.1**](https://www.ncbi.nlm.nih.gov/nuccore/AF409137.1)

**3.** **AF409138.1:** [**https://www.ncbi.nlm.nih.gov/nuccore/AF409138.1**](https://www.ncbi.nlm.nih.gov/nuccore/AF409138.1)

**4.** **HB977629.1:** [**https://www.ncbi.nlm.nih.gov/nuccore/HB977629.1**](https://www.ncbi.nlm.nih.gov/nuccore/HB977629.1)

**5.** **KX683219.1:** [**https://www.ncbi.nlm.nih.gov/nuccore/KX683219.1**](https://www.ncbi.nlm.nih.gov/nuccore/KX683219.1)

**6.** **KX764643.1:** [**https://www.ncbi.nlm.nih.gov/nuccore/KX764643.1**](https://www.ncbi.nlm.nih.gov/nuccore/KX764643.1)

**7.** **KX764644.1:** [**https://www.ncbi.nlm.nih.gov/nuccore/KX764644.1**](https://www.ncbi.nlm.nih.gov/nuccore/KX764644.1)

**8.** **KX764645.1:** [**https://www.ncbi.nlm.nih.gov/nuccore/KX764645.1**](https://www.ncbi.nlm.nih.gov/nuccore/KX764645.1)

**9.** **KX894508.1:** [**https://www.ncbi.nlm.nih.gov/nuccore/KX894508.1**](https://www.ncbi.nlm.nih.gov/nuccore/KX894508.1)

**10.** **KY702007.1:** [**https://www.ncbi.nlm.nih.gov/nuccore/KY702007.1**](https://www.ncbi.nlm.nih.gov/nuccore/KY702007.1)

**11.** **KY829023.3:** [**https://www.ncbi.nlm.nih.gov/nuccore/KY829023.3**](https://www.ncbi.nlm.nih.gov/nuccore/KY829023.3)

**12.** **MG972412.1:** [**https://www.ncbi.nlm.nih.gov/nuccore/MG972412.1**](https://www.ncbi.nlm.nih.gov/nuccore/MG972412.1)

**13.** **MH646674.1:** [**https://www.ncbi.nlm.nih.gov/nuccore/MH646674.1**](https://www.ncbi.nlm.nih.gov/nuccore/MH646674.1)

**14.** **MH893760.2:** [**https://www.ncbi.nlm.nih.gov/nuccore/MH893760.2**](https://www.ncbi.nlm.nih.gov/nuccore/MH893760.2)

**15.** **MK441838.1:** [**https://www.ncbi.nlm.nih.gov/nuccore/MK441838.1**](https://www.ncbi.nlm.nih.gov/nuccore/MK441838.1)

**16.** **MN072619.1:** [**https://www.ncbi.nlm.nih.gov/nuccore/MN072619.1**](https://www.ncbi.nlm.nih.gov/nuccore/MN072619.1)

**17.** **MN636838.1:** [**https://www.ncbi.nlm.nih.gov/nuccore/MN636838.1**](https://www.ncbi.nlm.nih.gov/nuccore/MN636838.1)

**18.** **MN636839.1:** [**https://www.ncbi.nlm.nih.gov/nuccore/MN636839.1**](https://www.ncbi.nlm.nih.gov/nuccore/MN636839.1)

**19.** **MN636840.1:** [**https://www.ncbi.nlm.nih.gov/nuccore/MN636840.1**](https://www.ncbi.nlm.nih.gov/nuccore/MN636840.1)

**20.** **MN636841.1:** [**https://www.ncbi.nlm.nih.gov/nuccore/MN636841.1**](https://www.ncbi.nlm.nih.gov/nuccore/MN636841.1)

**21.** **MN636842.1:** [**https://www.ncbi.nlm.nih.gov/nuccore/MN636842.1**](https://www.ncbi.nlm.nih.gov/nuccore/MN636842.1)

**22.** **MN636843.1:** [**https://www.ncbi.nlm.nih.gov/nuccore/MN636843.1**](https://www.ncbi.nlm.nih.gov/nuccore/MN636843.1)

**23.** **MN642592.1:** [**https://www.ncbi.nlm.nih.gov/nuccore/MN642592.1**](https://www.ncbi.nlm.nih.gov/nuccore/MN642592.1)

**24.** **MN995838.1:** [**https://www.ncbi.nlm.nih.gov/nuccore/MN995838.1**](https://www.ncbi.nlm.nih.gov/nuccore/MN995838.1)

**25.** **MT130502.2:** [**https://www.ncbi.nlm.nih.gov/nuccore/MT130502.2**](https://www.ncbi.nlm.nih.gov/nuccore/MT130502.2)

**26.** **MT992618.1:** [**https://www.ncbi.nlm.nih.gov/nuccore/MT992618.1**](https://www.ncbi.nlm.nih.gov/nuccore/MT992618.1)

**27.** **NC_003027.1:** [**https://www.ncbi.nlm.nih.gov/nuccore/NC_003027.1**](https://www.ncbi.nlm.nih.gov/nuccore/NC_003027.1)

**Supplementary File 1: B.** Multiple alignment of LSDV031 from 27 isolates of LSDV

AF325528.1_LSDV031 MAAYNSQYYANTPFYITTKEGKYLVLKAIKVCDIRTVECDGDKASCILKVEKSNQTCDRP 60

AF409137.1_LSDV031 MAAYNSQYYANTPFYITTKEGKYLVLKAIKVCDIRTVECDGDKASCILKVEKSNQTCDRP 60

AF409138.1_LSDV031 MAAYNSQYYANTPFYITTKEGKYLVLKAIKVCDIRTVECDGDKASCILKVEKSNQTCDRP 60

HB977629.1_LSDV031 MAAYNSQYYANTPFYITTKEGKYLVLKAIKVCDIRTVECDGDKASCILKVEKSNQTCDRP 60

KX683219.1_LSDV031 MAAYNSQYYANTPFYITTKEGKYLVLKAIKVCDIRTVECDGDKASCILKVEKSNQTCDRP 60

KX764643.1_LSDV031 MAAYNSQYYANTPFYITTKEGKYLVLKAIKVCDIRTVECDGDKASCILKVEKSNQTCDRP 60

KX764644.1_LSDV031 MAAYNSQYYANTPFYITTKEGKYLVLKAIKVCDIRTVECDGDKASCILKVEKSNQTCDRP 60

KX764645.1_LSDV031 MAAYNSQYYANTPFYITTKEGKYLVLKAIKVCDIRTVECDGDKASCILKVEKSNQTCDRP 60

KX894508.1_LSDV031 MAAYNSQYYANTPFYITTKEGKYLVLKAIKVCDIRTVECDGDKASCILKVEKSNQTCDRP 60

KY702007.1_LSDV031 MAAYNSQYYANTPFYITTKEGKYLVLKAIKVCDIRTVECDGDKASCILKVEKSNQTCDRP 60

KY829023.3_LSDV031 MAAYNSQYYANTPFYITTKEGKYLVLKAIKVCDIRTVECDGDKASCILKVEKSNQTCDRP 60

MG972412.1_LSDV031 MAAYNSQYYANTPFYITTKEGKYLVLKAIKVCDIRTVECDGDKASCILKVEKSNQTCDRP 60

MH646674.1_LSDV031 MAAYNSQYYANTPFYITTKEGKYLVLKAIKVCDIRTVECDGDKASCILKVEKSNQTCDRP 60

MH893760.2_LSDV031 MAAYNSQYYANTPFYITTKEGKYLVLKAIKVCDIRTVECDGDKASCILKVEKSNQTCDRP 60

MK441838.1_LSDV031 MAAYNSQYYANTPFYITTKEGKYLVLKAIKVCDIRTVECDGDKASCILKVEKSNQTCDRP 60

MN072619.1_LSDV031 MAAYNSQYYANTPFYITTKEGKYLVLKAIKVCDIRTVECDGDKASCILKVEKSNQTCDRP 60

MN636838.1_LSDV031 MAAYNSQYYANTPFYITTKEGKYLVLKAIKVCDIRTVECDGDKASCILKVEKSNQTCDRP 60

MN636839.1_LSDV031 MAAYNSQYYANTPFYITTKEGKYLVLKAIKVCDIRTVECDGDKASCILKVEKSNQTCDRP 60

MN636840.1_LSDV031 MAAYNSQYYANTPFYITTKEGKYLVLKAIKVCDIRTVECDGDKASCILKVEKSNQTCDRP 60

MN636841.1_LSDV031 MAAYNSQYYANTPFYITTKEGKYLVLKAIKVCDIRTVECDGDKASCILKVEKSNQTCDRP 60

MN636842.1_LSDV031 MAAYNSQYYANTPFYITTKEGKYLVLKAIKVCDIRTVECDGDKASCILKVEKSNQTCDRP 60

MN636843.1_LSDV031 MAAYNSQYYANTPFYITTKEGKYLVLKAIKVCDIRTVECDGDKASCILKVEKSNQTCDRP 60

MN642592.1_LSDV031 MAAYNSQYYANTPFYITTKEGKYLVLKAIKVCDIRTVECDGDKASCILKVEKSNQTCDRP 60

MN995838.1_LSDV031 MAAYNSQYYANTPFYITTKEGKYLVLKAIKVCDIRTVECDGDKASCILKVEKSNQTCDRP 60

MT130502.2_LSDV031 MAAYNSQYYANTPFYITTKEGKYLVLKAIKVCDIRTVECDGDKASCILKVEKSNQTCDRP 60

MT992618.1_LSDV031 MAAYNSQYYANTPFYITTKEGKYLVLKAIKVCDIRTVECDGDKASCILKVEKSNQTCDRP 60

NC_003027.1_LSDV031 MAAYNSQYYANTPFYITTKEGKYLVLKAIKVCDIRTVECDGDKASCILKVEKSNQTCDRP 60

************************************************************

AF325528.1_LSDV031 SSPCERAARASSPNRGNNSVPFMRTNMLEDLQAKNRSVMSRILG 104

AF409137.1_LSDV031 SSPCERAARASSPNRGNNSVPFMRTNMLEDLQAKNRSVMSRILG 104

AF409138.1_LSDV031 SSPCERAARASSPNRGNNSVPFMRTNMLEDLQAKNRSVMSRILG 104

HB977629.1_LSDV031 SSPCERAARASSPNRGNNSVPFMRTNMLEDLQAKNRSVMSRILG 104

KX683219.1_LSDV031 SSPCERAARASSPNRGNNSVPFMRTNMLEDLQAKNRSVMSRILG 104

KX764643.1_LSDV031 SSPCERAARASSPNRGNNSVPFMRTNMLEDLQAKNRSVMSRILG 104

KX764644.1_LSDV031 SSPCERAARASSPNRGNNSVPFMRTNMLEDLQAKNRSVMSRILG 104

KX764645.1_LSDV031 SSPCERAARASSPNRGNNSVPFMRTNMLEDLQAKNRSVMSRILG 104

KX894508.1_LSDV031 SSPCERAARASSPNRGNNSVPFMRTNMLEDLQAKNRSVMSRILG 104

KY702007.1_LSDV031 SSPCERAARASSPNRGNNSVPFMRTNMLEDLQAKNRSVMSRILG 104

KY829023.3_LSDV031 SSPCERAARASSPNRGNNSVPFMRTNMLEDLQAKNRSVMSRILG 104

MG972412.1_LSDV031 SSPCERAARASSPNRGNNSVPFMRTNMLEDLQAKNRSVMSRILG 104

MH646674.1_LSDV031 SSPCERAARASSPNRGNNSVPFMRTNMLEDLQAKNRSVMSRILG 104

MH893760.2_LSDV031 SSPCERAARASSPNRGNNSVPFMRTNMLEDLQAKNRSVMSRILG 104

MK441838.1_LSDV031 SSPCERAARASSPNRGNNSVPFMRTNMLEDLQAKNRSVMSRILG 104

MN072619.1_LSDV031 SSPCERAARASSPNRGNNSVPFMRTNMLEDLQAKNRSVMSRILG 104

MN636838.1_LSDV031 SSPCERAARASSPNRGNNSVPFMRTNMLEDLQAKNRSVMSRILG 104

MN636839.1_LSDV031 SSPCERAARASSPNRGNNSVPFMRTNMLEDLQAKNRSVMSRILG 104

MN636840.1_LSDV031 SSPCERAARASSPNRGNNSVPFMRTNMLEDLQAKNRSVMSRILG 104

MN636841.1_LSDV031 SSPCERAARASSPNRGNNSVPFMRTNMLEDLQAKNRSVMSRILG 104

MN636842.1_LSDV031 SSPCERAARASSPNRGNNSVPFMRTNMLEDLQAKNRSVMSRILG 104

MN636843.1_LSDV031 SSPCERAARASSPNRGNNSVPFMRTNMLEDLQAKNRSVMSRILG 104

MN642592.1_LSDV031 SSPCERAARASSPNRGNNSVPFMRTNMLEDLQAKNRSVMSRILG 104

MN995838.1_LSDV031 SSPCERAARASSPNRGNNSVPFMRTNMLEDLQAKNRSVMSRILG 104

MT130502.2_LSDV031 SSPCERAARASSPNRGNNSVPFMRTNMLEDLQAKNRSVMSRILG 104

MT992618.1_LSDV031 SSPCERAARASSPNRGNNSVPFMRTNMLEDLQAKNRSVMSRILG 104

NC_003027.1_LSDV031 SSPCERAARASSPNRGNNSVPFMRTNMLEDLQAKNRSVMSRILG 104

********************************************

**Multiple alignment of LSDV090 from 27 isolates of LSDV**

AF325528.1_LSDV090 MNNTVINSLIGNDDNIKRHNVFGVDIQNPTLYMPQYITLNGVSSTGSCTQNVVSTFEIRD 60

AF409137.1_LSDV090 MNNTVINSLIGNDDNIKRHNVFGVDIQNPTLYMPQYITLNGVSSTGSCTQNVVSTFEIRD 60

KX683219.1_LSDV090 MNNTVINSLIGNDDNIKRHNVFGVDIQNPTLYMPQYITLNGVSSTGSCTQNVVSTFEIRD 60

KX894508.1_LSDV090 MNNTVINSLIGNDDNIKRHNVFGVDIQNPTLYMPQYITLNGVSSTGSCTQNVVSTFEIRD 60

KY702007.1_LSDV090 MNNTVINSLIGNDDNIKRHNVFGVDIQNPTLYMPQYITLNGVSSTGSCTQNVVSTFEIRD 60

KY829023.3_LSDV090 MNNTVINSLIGNDDNIKRHNVFGVDIQNPTLYMPQYITLNGVSSTGSCTQNVVSTFEIRD 60

MH893760.2_LSDV090 MNNTVINSLIGNDDNIKRHNVFGVDIQNPTLYMPQYITLNGVSSTGSCTQNVVSTFEIRD 60

MN072619.1_LSDV090 MNNTVINSLIGNDDNIKRHNVFGVDIQNPTLYMPQYITLNGVSSTGSCTQNVVSTFEIRD 60

MN642592.1_LSDV090 MNNTVINSLIGNDDNIKRHNVFGVDIQNPTLYMPQYITLNGVSSTGSCTQNVVSTFEIRD 60

MN995838.1_LSDV090 MNNTVINSLIGNDDNIKRHNVFGVDIQNPTLYMPQYITLNGVSSTGSCTQNVVSTFEIRD 60

MT130502.2_LSDV090 MNNTVINSLIGNDDNIKRHNVFGVDIQNPTLYMPQYITLNGVSSTGSCTQNVVSTFEIRD 60

NC_003027.1_LSDV090 MNNTVINSLIGNDDNIKRHNVFGVDIQNPTLYMPQYITLNGVSSTGSCTQNVVSTFEIRD 60

AF409138.1_LSDV090 MNNTVINSLIGNDDNIKRHNVFGVDIQNPTLYMPQYITLNGVSSTGSCTQNVVSTFEIRD 60

HB977629.1_LSDV090 MNNTVINSLIGNDDNIKRHNVFGVDIQNPTLYMPQYITLNGVSSTGSCTQNVVSTFEIRD 60

KX764643.1_LSDV090 MNNTVINSLIGNDDNIKRHNVFGVDIQNPTLYMPQYITLNGVSSTGSCTQNVVSTFEIRD 60

KX764644.1_LSDV090 MNNTVINSLIGNDDNIKRHNVFGVDIQNPTLYMPQYITLNGVSSTGSCTQNVVSTFEIRD 60

KX764645.1_LSDV090 MNNTVINSLIGNDDNIKRHNVFGVDIQNPTLYMPQYITLNGVSSTGSCTQNVVSTFEIRD 60

MG972412.1_LSDV090 MNNTVINSLIGNDDNIKRHNVFGVDIQNPTLYMPQYITLNGVSSTGSCTQNVVSTFEIRD 60

MH646674.1_LSDV090 MNNTVINSLIGNDDNIKRHNVFGVDIQNPTLYMPQYITLNGVSSTGSCTQNVVSTFEIRD 60

MK441838.1_LSDV090 MNNTVINSLIGNDDNIKRHNVFGVDIQNPTLYMPQYITLNGVSSTGSCTQNVVSTFEIRD 60

MN636838.1_LSDV090 MNNTVINSLIGNDDNIKRHNVFGVDIQNPTLYMPQYITLNGVSSTGSCTQNVVSTFEIRD 60

MN636839.1_LSDV090 MNNTVINSLIGNDDNIKRHNVFGVDIQNPTLYMPQYITLNGVSSTGSCTQNVVSTFEIRD 60

MN636840.1_LSDV090 MNNTVINSLIGNDDNIKRHNVFGVDIQNPTLYMPQYITLNGVSSTGSCTQNVVSTFEIRD 60

MN636841.1_LSDV090 MNNTVINSLIGNDDNIKRHNVFGVDIQNPTLYMPQYITLNGVSSTGSCTQNVVSTFEIRD 60

MN636842.1_LSDV090 MNNTVINSLIGNDDNIKRHNVFGVDIQNPTLYMPQYITLNGVSSTGSCTQNVVSTFEIRD 60

MN636843.1_LSDV090 MNNTVINSLIGNDDNIKRHNVFGVDIQNPTLYMPQYITLNGVSSTGSCTQNVVSTFEIRD 60

MT992618.1_LSDV090 MNNTVINSLIGNDDNIKRHNVFGVDIQNPTLYMPQYITLNGVSSTGSCTQNVVSTFEIRD 60

************************************************************

AF325528.1_LSDV090 QYITALSHLMLSIDLPEVKGIGKFGYVTYVGYKSIQHVSISCNNGTIWESSGEDLFYSCK 120

AF409137.1_LSDV090 QYITALSHLMLSIDLPEVKGIGKFGYVTYVGYKSIQHVSISCNNGTIWESSGEDLFYSCK 120

KX683219.1_LSDV090 QYITALSHLMLSIDLPEVKGIGKFGYVTYVGYKSIQHVSISCNNGTIWESSGEDLFYSCK 120

KX894508.1_LSDV090 QYITALSHLMLSIDLPEVKGIGKFGYVTYVGYKSIQHVSISCNNGTIWESSGEDLFYSCK 120

KY702007.1_LSDV090 QYITALSHLMLSIDLPEVKGIGKFGYVTYVGYKSIQHVSISCNNGTIWESSGEDLFYSCK 120

KY829023.3_LSDV090 QYITALSHLMLSIDLPEVKGIGKFGYVTYVGYKSIQHVSISCNNGTIWESSGEDLFYSCK 120

MH893760.2_LSDV090 QYITALSHLMLSIDLPEVKGIGKFGYVTYVGYKSIQHVSISCNNGTIWESSGEDLFYSCK 120

MN072619.1_LSDV090 QYITALSHLMLSIDLPEVKGIGKFGYVTYVGYKSIQHVSISCNNGTIWESSGEDLFYSCK 120

MN642592.1_LSDV090 QYITALSHLMLSIDLPEVKGIGKFGYVTYVGYKSIQHVSISCNNGTIWESSGEDLFYSCK 120

MN995838.1_LSDV090 QYITALSHLMLSIDLPEVKGIGKFGYVTYVGYKSIQHVSISCNNGTIWESSGEDLFYSCK 120

MT130502.2_LSDV090 QYITALSHLMLSIDLPEVKGIGKFGYVTYVGYKSIQHVSISCNNGTIWESSGEDLFYSCK 120

NC_003027.1_LSDV090 QYITALSHLMLSIDLPEVKGIGKFGYVTYVGYKSIQHVSISCNNGTIWESSGEDLFYSCK 120

AF409138.1_LSDV090 QYITALSHLMLSIDLPEVKGIGKFGYVTYVGYKSIQHVSISCNNGTIWESSGEDLFYSCK 120

HB977629.1_LSDV090 QYITALSHLMLSIDLPEVKGIGKFGYVTYVGYKSIQHVSISCNNGTIWESSGEDLFYSCK 120

KX764643.1_LSDV090 QYITALSHLMLSIDLPEVKGIGKFGYVTYVGYKSIQHVSISCNNGTIWESSGEDLFYSCK 120

KX764644.1_LSDV090 QYITALSHLMLSIDLPEVKGIGKFGYVTYVGYKSIQHVSISCNNGTIWESSGEDLFYSCK 120

KX764645.1_LSDV090 QYITALSHLMLSIDLPEVKGIGKFGYVTYVGYKSIQHVSISCNNGTIWESSGEDLFYSCK 120

MG972412.1_LSDV090 QYITALSHLMLSIDLPEVKGIGKFGYVTYVGYKSIQHVSISCNNGTIWESSGEDLFYSCK 120

MH646674.1_LSDV090 QYITALSHLMLSIDLPEVKGIGKFGYVTYVGYKSIQHVSISCNNGTIWESSGEDLFYSCK 120

MK441838.1_LSDV090 QYITALSHLMLSIDLPEVKGIGKFGYVTYVGYKSIQHVSISCNNGTIWESSGEDLFYSCK 120

MN636838.1_LSDV090 QYITALSHLMLSIDLPEVKGIGKFGYVTYVGYKSIQHVSISCNNGTIWESSGEDLFYSCK 120

MN636839.1_LSDV090 QYITALSHLMLSIDLPEVKGIGKFGYVTYVGYKSIQHVSISCNNGTIWESSGEDLFYSCK 120

MN636840.1_LSDV090 QYITALSHLMLSIDLPEVKGIGKFGYVTYVGYKSIQHVSISCNNGTIWESSGEDLFYSCK 120

MN636841.1_LSDV090 QYITALSHLMLSIDLPEVKGIGKFGYVTYVGYKSIQHVSISCNNGTIWESSGEDLFYSCK 120

MN636842.1_LSDV090 QYITALSHLMLSIDLPEVKGIGKFGYVTYVGYKSIQHVSISCNNGTIWESSGEDLFYSCK 120

MN636843.1_LSDV090 QYITALSHLMLSIDLPEVKGIGKFGYVTYVGYKSIQHVSISCNNGTIWESSGEDLFYSCK 120

MT992618.1_LSDV090 QYITALSHLMLSIDLPEVKGIGKFGYVTYVGYKSIQHVSISCNNGTIWESSGEDLFYSCK 120

************************************************************

AF325528.1_LSDV090 NNETALNNSGFCHELNSISTGLTNNDTIKESATIYVYIKTPFDMEKTFSSLKLSDSKVIV 180

AF409137.1_LSDV090 NNETALNNSGFCHELNSISTGLTNNDTIKESATIYVYIKTPFDMEKTFSSLKLSDSKVIV 180

KX683219.1_LSDV090 NNETALNNSGFCHELNSISTGLTNNDTIKESATIYVYIKTPFDMEKTFSSLKLSDSKVIV 180

KX894508.1_LSDV090 NNETALNNSGFCHELNSISTGLTNNDTIKESATIYVYIKTPFDMEKTFSSLKLSDSKVIV 180

KY702007.1_LSDV090 NNETALNNSGFCHELNSISTGLTNNDTIKESATIYVYIKTPFDMEKTFSSLKLSDSKVIV 180

KY829023.3_LSDV090 NNETALNNSGFCHELNSISTGLTNNDTIKESATIYVYIKTPFDMEKTFSSLKLSDSKVIV 180

MH893760.2_LSDV090 NNETALNNSGFCHELNSISTGLTNNDTIKESATIYVYIKTPFDMEKTFSSLKLSDSKVIV 180

MN072619.1_LSDV090 NNETALNNSGFCHELNSISTGLTNNDTIKESATIYVYIKTPFDMEKTFSSLKLSDSKVIV 180

MN642592.1_LSDV090 NNETALNNSGFCHELNSISTGLTNNDTIKESATIYVYIKTPFDMEKTFSSLKLSDSKVIV 180

MN995838.1_LSDV090 NNETALNNSGFCHELNSISTGLTNNDTIKESATIYVYIKTPFDMEKTFSSLKLSDSKVIV 180

MT130502.2_LSDV090 NNETALNNSGFCHELNSISTGLTNNDTIKESATIYVYIKTPFDMEKTFSSLKLSDSKVIV 180

NC_003027.1_LSDV090 NNETALNNSGFCHELNSISTGLTNNDTIKESATIYVYIKTPFDMEKTFSSLKLSDSKVIV 180

AF409138.1_LSDV090 NNETALNNSGFCHELNSISTGLTNNDTIKESATIYVYIKTPFDMEKTFSSLKLSDSKVIV 180

HB977629.1_LSDV090 NNETALNNSGFCHELNSISTGLTNNDTIKESATIYVYIKTPFDMEKTFSSLKLSDSKVIV 180

KX764643.1_LSDV090 NNETALNNSGFCHELNSISTGLTNNDTIKESATIYVYIKTPFDMEKTFSSLKLSDSKVIV 180

KX764644.1_LSDV090 NNETALNNSGFCHELNSISTGLTNNDTIKESATIYVYIKTPFDMEKTFSSLKLSDSKVIV 180

KX764645.1_LSDV090 NNETALNNSGFCHELNSISTGLTNNDTIKESATIYVYIKTPFDMEKTFSSLKLSDSKVIV 180

MG972412.1_LSDV090 NNETALNNSGFCHELNSISTGLTNNDTIKESATIYVYIKTPFDMEKTFSSLKLSDSKVIV 180

MH646674.1_LSDV090 NNETALNNSGFCHELNSISTGLTNNDTIKESATIYVYIKTPFDMEKTFSSLKLSDSKVIV 180

MK441838.1_LSDV090 NNETALNNSGFCHELNSISTGLTNNDTIKESATIYVYIKTPFDMEKTFSSLKLSDSKVIV 180

MN636838.1_LSDV090 NNETALNNSGFCHELNSISTGLTNNDTIKESATIYVYIKTPFDMEKTFSSLKLSDSKVIV 180

MN636839.1_LSDV090 NNETALNNSGFCHELNSISTGLTNNDTIKESATIYVYIKTPFDMEKTFSSLKLSDSKVIV 180

MN636840.1_LSDV090 NNETALNNSGFCHELNSISTGLTNNDTIKESATIYVYIKTPFDMEKTFSSLKLSDSKVIV 180

MN636841.1_LSDV090 NNETALNNSGFCHELNSISTGLTNNDTIKESATIYVYIKTPFDMEKTFSSLKLSDSKVIV 180

MN636842.1_LSDV090 NNETALNNSGFCHELNSISTGLTNNDTIKESATIYVYIKTPFDMEKTFSSLKLSDSKVIV 180

MN636843.1_LSDV090 NNETALNNSGFCHELNSISTGLTNNDTIKESATIYVYIKTPFDMEKTFSSLKLSDSKVIV 180

MT992618.1_LSDV090 NNETALNNSGFCHELNSISTGLTNNDTIKESATIYVYIKTPFDMEKTFSSLKLSDSKVIV 180

************************************************************

AF325528.1_LSDV090 TITFNPVSDIIIRDSTFDYESFVKDFIYVTELSFIGYMVKNIQTKESYIEIPRRVLGQIN 240

AF409137.1_LSDV090 TITFNPVSDIIIRDSTFDYESFVKDFIYVTELSFIGYMVKNIQTKESYIEIPRRVLGQIN 240

KX683219.1_LSDV090 TITFNPVSDIIIRDSTFDYESFVKDFIYVTELSFIGYMVKNIQTKESYIEIPRRVLGQIN 240

KX894508.1_LSDV090 TITFNPVSDIIIRDSTFDYESFVKDFIYVTELSFIGYMVKNIQTKESYIEIPRRVLGQIN 240

KY702007.1_LSDV090 TITFNPVSDIIIRDSTFDYESFVKDFIYVTELSFIGYMVKNIQTKESYIEIPRRVLGQIN 240

KY829023.3_LSDV090 TITFNPVSDIIIRDSTFDYESFVKDFIYVTELSFIGYMVKNIQTKESYIEIPRRVLGQIN 240

MH893760.2_LSDV090 TITFNPVSDIIIRDSTFDYESFVKDFIYVTELSFIGYMVKNIQTKESYIEIPRRVLGQIN 240

MN072619.1_LSDV090 TITFNPVSDIIIRDSTFDYESFVKDFIYVTELSFIGYMVKNIQTKESYIEIPRRVLGQIN 240

MN642592.1_LSDV090 TITFNPVSDIIIRDSTFDYESFVKDFIYVTELSFIGYMVKNIQTKESYIEIPRRVLGQIN 240

MN995838.1_LSDV090 TITFNPVSDIIIRDSTFDYESFVKDFIYVTELSFIGYMVKNIQTKESYIEIPRRVLGQIN 240

MT130502.2_LSDV090 TITFNPVSDIIIRDSTFDYESFVKDFIYVTELSFIGYMVKNIQTKESYIEIPRRVLGQIN 240

NC_003027.1_LSDV090 TITFNPVSDIIIRDSTFDYESFVKDFIYVTELSFIGYMVKNIQTKESYIEIPRRVLGQIN 240

AF409138.1_LSDV090 TITFNPVSDIIIRDSTFDYESFVKDFIYVTELSFIGYMVKNIQTKESYIEIPRRVLGQIN 240

HB977629.1_LSDV090 TITFNPVSDIIIRDSTFDYESFVKDFIYVTELSFIGYMVKNIQTKESYIEIPRRVLGQIN 240

KX764643.1_LSDV090 TITFNPVSDIIIRDSTFDYESFVKDFIYVTELSFIGYMVKNIQTKESYIEIPRRVLGQIN 240

KX764644.1_LSDV090 TITFNPVSDIIIRDSTFDYESFVKDFIYVTELSFIGYMVKNIQTKESYIEIPRRVLGQIN 240

KX764645.1_LSDV090 TITFNPVSDIIIRDSTFDYESFVKDFIYVTELSFIGYMVKNIQTKESYIEIPRRVLGQIN 240

MG972412.1_LSDV090 TITFNPVSDIIIRDSTFDYESFVKDFIYVTELSFIGYMVKNIQTKESYIEIPRRVLGQIN 240

MH646674.1_LSDV090 TITFNPVSDIIIRDSTFDYESFVKDFIYVTELSFIGYMVKNIQTKESYIEIPRRVLGQIN 240

MK441838.1_LSDV090 TITFNPVSDIIIRDSTFDYESFVKDFIYVTELSFIGYMVKNIQTKESYIEIPRRVLGQIN 240

MN636838.1_LSDV090 TITFNPVSDIIIRDSTFDYESFVKDFIYVTELSFIGYMVKNIQTKESYIEIPRRVLGQIN 240

MN636839.1_LSDV090 TITFNPVSDIIIRDSTFDYESFVKDFIYVTELSFIGYMVKNIQTKESYIEIPRRVLGQIN 240

MN636840.1_LSDV090 TITFNPVSDIIIRDSTFDYESFVKDFIYVTELSFIGYMVKNIQTKESYIEIPRRVLGQIN 240

MN636841.1_LSDV090 TITFNPVSDIIIRDSTFDYESFVKDFIYVTELSFIGYMVKNIQTKESYIEIPRRVLGQIN 240

MN636842.1_LSDV090 TITFNPVSDIIIRDSTFDYESFVKDFIYVTELSFIGYMVKNIQTKESYIEIPRRVLGQIN 240

MN636843.1_LSDV090 TITFNPVSDIIIRDSTFDYESFVKDFIYVTELSFIGYMVKNIQTKESYIEIPRRVLGQIN 240

MT992618.1_LSDV090 TITFNPVSDIIIRDSTFDYESFVKDFIYVTELSFIGYMVKNIQTKESYIEIPRRVLGQIN 240

************************************************************

AF325528.1_LSDV090 QSTAVISEINSVTSFSVYVKPYYGNTDNKFIAYPGYTQSERDFICVFVERLLEDLVVVSK 300

AF409137.1_LSDV090 QSTAVISEINSVTSFSVYVKPYYGNTDNKFIAYPGYTQSERDFICVFVERLLEDLVVVSK 300

KX683219.1_LSDV090 QSTAVISEINSVTSFSVYVKPYYGNTDNKFIAYPGYTQSERDFICVFVERLLEDLVVVSK 300

KX894508.1_LSDV090 QSTAVISEINSVTSFSVYVKPYYGNTDNKFIAYPGYTQSERDFICVFVERLLEDLVVVSK 300

KY702007.1_LSDV090 QSTAVISEINSVTSFSVYVKPYYGNTDNKFIAYPGYTQSERDFICVFVERLLEDLVVVSK 300

KY829023.3_LSDV090 QSTAVISEINSVTSFSVYVKPYYGNTDNKFIAYPGYTQSERDFICVFVERLLEDLVVVSK 300

MH893760.2_LSDV090 QSTAVISEINSVTSFSVYVKPYYGNTDNKFIAYPGYTQSERDFICVFVERLLEDLVVVSK 300

MN072619.1_LSDV090 QSTAVISEINSVTSFSVYVKPYYGNTDNKFIAYPGYTQSERDFICVFVERLLEDLVVVSK 300

MN642592.1_LSDV090 QSTAVISEINSVTSFSVYVKPYYGNTDNKFIAYPGYTQSERDFICVFVERLLEDLVVVSK 300

MN995838.1_LSDV090 QSTAVISEINSVTSFSVYVKPYYGNTDNKFIAYPGYTQSERDFICVFVERLLEDLVVVSK 300

MT130502.2_LSDV090 QSTAVISEINSVTSFSVYVKPYYGNTDNKFIAYPGYTQSERDFICVFVERLLEDLVVVSK 300

NC_003027.1_LSDV090 QSTAVISEINSVTSFSVYVKPYYGNTDNKFIAYPGYTQSERDFICVFVERLLEDLVVVSK 300

AF409138.1_LSDV090 QSTAVISEINSVTSFSVYVKPYYGNTDNKFIAYPGYsQSERDFICVFVERLLEDLVVVSK 300

HB977629.1_LSDV090 QSTAVISEINSVTSFSVYVKPYYGNTDNKFIAYPGYsQSERDFICVFVERLLEDLVVVSK 300

KX764643.1_LSDV090 QSTAVISEINSVTSFSVYVKPYYGNTDNKFIAYPGYsQSERDFICVFVERLLEDLVVVSK 300

KX764644.1_LSDV090 QSTAVISEINSVTSFSVYVKPYYGNTDNKFIAYPGYsQSERDFICVFVERLLEDLVVVSK 300

KX764645.1_LSDV090 QSTAVISEINSVTSFSVYVKPYYGNTDNKFIAYPGYsQSERDFICVFVERLLEDLVVVSK 300

MG972412.1_LSDV090 QSTAVISEINSVTSFSVYVKPYYGNTDNKFIAYPGYsQSERDFICVFVERLLEDLVVVSK 300

MH646674.1_LSDV090 QSTAVISEINSVTSFSVYVKPYYGNTDNKFIAYPGYsQSERDFICVFVERLLEDLVVVSK 300

MK441838.1_LSDV090 QSTAVISEINSVTSFSVYVKPYYGNTDNKFIAYPGYsQSERDFICVFVERLLEDLVVVSK 300

MN636838.1_LSDV090 QSTAVISEINSVTSFSVYVKPYYGNTDNKFIAYPGYsQSERDFICVFVERLLEDLVVVSK 300

MN636839.1_LSDV090 QSTAVISEINSVTSFSVYVKPYYGNTDNKFIAYPGYsQSERDFICVFVERLLEDLVVVSK 300

MN636840.1_LSDV090 QSTAVISEINSVTSFSVYVKPYYGNTDNKFIAYPGYsQSERDFICVFVERLLEDLVVVSK 300

MN636841.1_LSDV090 QSTAVISEINSVTSFSVYVKPYYGNTDNKFIAYPGYsQSERDFICVFVERLLEDLVVVSK 300

MN636842.1_LSDV090 QSTAVISEINSVTSFSVYVKPYYGNTDNKFIAYPGYsQSERDFICVFVERLLEDLVVVSK 300

MN636843.1_LSDV090 QSTAVISEINSVTSFSVYVKPYYGNTDNKFIAYPGYsQSERDFICVFVERLLEDLVVVSK 300

MT992618.1_LSDV090 QSTAVISEINSVTSFSVYVKPYYGNTDNKFIAYPGYsQSERDFICVFVERLLEDLVVVSK 300

************************************:***********************

AF325528.1_LSDV090 EPPNYFPESAEFIEVPPNGIVNIQDVDVFIKIDNVPCGMSIYYHSNILVFGTRKNSITYN 360

AF409137.1_LSDV090 EPPNYFPESAEFIEVPPNGIVNIQDVDVFIKIDNVPCGMSIYYHSNILVFGTRKNSITYN 360

KX683219.1_LSDV090 EPPNYFPESAEFIEVPPNGIVNIQDVDVFIKIDNVPCGMSIYYHSNILVFGTRKNSITYN 360

KX894508.1_LSDV090 EPPNYFPESAEFIEVPPNGIVNIQDVDVFIKIDNVPCGMSIYYHSNILVFGTRKNSITYN 360

KY702007.1_LSDV090 EPPNYFPESAEFIEVPPNGIVNIQDVDVFIKIDNVPCGMSIYYHSNILVFGTRKNSITYN 360

KY829023.3_LSDV090 EPPNYFPESAEFIEVPPNGIVNIQDVDVFIKIDNVPCGMSIYYHSNILVFGTRKNSITYN 360

MH893760.2_LSDV090 EPPNYFPESAEFIEVPPNGIVNIQDVDVFIKIDNVPCGMSIYYHSNILVFGTRKNSITYN 360

MN072619.1_LSDV090 EPPNYFPESAEFIEVPPNGIVNIQDVDVFIKIDNVPCGMSIYYHSNILVFGTRKNSITYN 360

MN642592.1_LSDV090 EPPNYFPESAEFIEVPPNGIVNIQDVDVFIKIDNVPCGMSIYYHSNILVFGTRKNSITYN 360

MN995838.1_LSDV090 EPPNYFPESAEFIEVPPNGIVNIQDVDVFIKIDNVPCGMSIYYHSNILVFGTRKNSITYN 360

MT130502.2_LSDV090 EPPNYFPESAEFIEVPPNGIVNIQDVDVFIKIDNVPCGMSIYYHSNILVFGTRKNSITYN 360

NC_003027.1_LSDV090 EPPNYFPESAEFIEVPPNGIVNIQDVDVFIKIDNVPCGMSIYYHSNILVFGTRKNSITYN 360

AF409138.1_LSDV090 EPPNYFPESAEFIEVPPNGIVNIQDVDVFIKIDNVPCGMSIYYHSNILVFGTRKNSITYN 360

HB977629.1_LSDV090 EPPNYFPESAEFIEVPPNGIVNIQDVDVFIKIDNVPCGMSIYYHSNILVFGTRKNSITYN 360

KX764643.1_LSDV090 EPPNYFPESAEFIEVPPNGIVNIQDVDVFIKIDNVPCGMSIYYHSNILVFGTRKNSITYN 360

KX764644.1_LSDV090 EPPNYFPESAEFIEVPPNGIVNIQDVDVFIKIDNVPCGMSIYYHSNILVFGTRKNSITYN 360

KX764645.1_LSDV090 EPPNYFPESAEFIEVPPNGIVNIQDVDVFIKIDNVPCGMSIYYHSNILVFGTRKNSITYN 360

MG972412.1_LSDV090 EPPNYFPESAEFIEVPPNGIVNIQDVDVFIKIDNVPCGMSIYYHSNILVFGTRKNSITYN 360

MH646674.1_LSDV090 EPPNYFPESAEFIEVPPNGIVNIQDVDVFIKIDNVPCGMSIYYHSNILVFGTRKNSITYN 360

MK441838.1_LSDV090 EPPNYFPESAEFIEVPPNGIVNIQDVDVFIKIDNVPCGMSIYYHSNILVFGTRKNSITYN 360

MN636838.1_LSDV090 EPPNYFPESAEFIEVPPNGIVNIQDVDVFIKIDNVPCGMSIYYHSNILVFGTRKNSITYN 360

MN636839.1_LSDV090 EPPNYFPESAEFIEVPPNGIVNIQDVDVFIKIDNVPCGMSIYYHSNILVFGTRKNSITYN 360

MN636840.1_LSDV090 EPPNYFPESAEFIEVPPNGIVNIQDVDVFIKIDNVPCGMSIYYHSNILVFGTRKNSITYN 360

MN636841.1_LSDV090 EPPNYFPESAEFIEVPPNGIVNIQDVDVFIKIDNVPCGMSIYYHSNILVFGTRKNSITYN 360

MN636842.1_LSDV090 EPPNYFPESAEFIEVPPNGIVNIQDVDVFIKIDNVPCGMSIYYHSNILVFGTRKNSITYN 360

MN636843.1_LSDV090 EPPNYFPESAEFIEVPPNGIVNIQDVDVFIKIDNVPCGMSIYYHSNILVFGTRKNSITYN 360

MT992618.1_LSDV090 EPPNYFPESAEFIEVPPNGIVNIQDVDVFIKIDNVPCGMSIYYHSNILVFGTRKNSITYN 360

************************************************************

AF325528.1_LSDV090 ISKKFSTITGSYSESTKRIMFSHISHSISITDVSIPVNLWSCQRNVYNGDNRSESSKNKD 420

AF409137.1_LSDV090 ISKKFSTITGSYSESTKRIMFSHISHSISITDVSIPVNLWSCQRNVYNGDNRSESSKNKD 420

KX683219.1_LSDV090 ISKKFSTITGSYSESTKRIMFSHISHSISITDVSIPVNLWSCQRNVYNGDNRSESSKNKD 420

KX894508.1_LSDV090 ISKKFSTITGSYSESTKRIMFSHISHSISITDVSIPVNLWSCQRNVYNGDNRSESSKNKD 420

KY702007.1_LSDV090 ISKKFSTITGSYSESTKRIMFSHISHSISITDVSIPVNLWSCQRNVYNGDNRSESSKNKD 420

KY829023.3_LSDV090 ISKKFSTITGSYSESTKRIMFSHISHSISITDVSIPVNLWSCQRNVYNGDNRSESSKNKD 420

MH893760.2_LSDV090 ISKKFSTITGSYSESTKRIMFSHISHSISITDVSIPVNLWSCQRNVYNGDNRSESSKNKD 420

MN072619.1_LSDV090 ISKKFSTITGSYSESTKRIMFSHISHSISITDVSIPVNLWSCQRNVYNGDNRSESSKNKD 420

MN642592.1_LSDV090 ISKKFSTITGSYSESTKRIMFSHISHSISITDVSIPVNLWSCQRNVYNGDNRSESSKNKD 420

MN995838.1_LSDV090 ISKKFSTITGSYSESTKRIMFSHISHSISITDVSIPVNLWSCQRNVYNGDNRSESSKNKD 420

MT130502.2_LSDV090 ISKKFSTITGSYSESTKRIMFSHISHSISITDVSIPVNLWSCQRNVYNGDNRSESSKNKD 420

NC_003027.1_LSDV090 ISKKFSTITGSYSESTKRIMFSHISHSISITDVSIPVNLWSCQRNVYNGDNRSESSKNKD 420

AF409138.1_LSDV090 ISKKFSTITGSYSESTKRIMFSHISHSISITDVSIPVNLWSCQRNVYNGDNRSESSKNKD 420

HB977629.1_LSDV090 ISKKFSTITGSYSESTKRIMFSHISHSISITDVSIPVNLWSCQRNVYNGDNRSESSKNKD 420

KX764643.1_LSDV090 ISKKFSTITGSYSESTKRIMFSHISHSISITDVSIPVNLWSCQRNVYNGDNRSESSKNKD 420

KX764644.1_LSDV090 ISKKFSTITGSYSESTKRIMFSHISHSISITDVSIPVNLWSCQRNVYNGDNRSESSKNKD 420

KX764645.1_LSDV090 ISKKFSTITGSYSESTKRIMFSHISHSISITDVSIPVNLWSCQRNVYNGDNRSESSKNKD 420

MG972412.1_LSDV090 ISKKFSTITGSYSESTKRIMFSHISHSISITDVSIPVNLWSCQRNVYNGDNRSESSKNKD 420

MH646674.1_LSDV090 ISKKFSTITGSYSESTKRIMFSHISHSISITDVSIPVNLWSCQRNVYNGDNRSESSKNKD 420

MK441838.1_LSDV090 ISKKFSTITGSYSESTKRIMFSHISHSISITDVSIPVNLWSCQRNVYNGDNRSESSKNKD 420

MN636838.1_LSDV090 ISKKFSTITGSYSESTKRIMFSHISHSISITDVSIPVNLWSCQRNVYNGDNRSESSKNKD 420

MN636839.1_LSDV090 ISKKFSTITGSYSESTKRIMFSHISHSISITDVSIPVNLWSCQRNVYNGDNRSESSKNKD 420

MN636840.1_LSDV090 ISKKFSTITGSYSESTKRIMFSHISHSISITDVSIPVNLWSCQRNVYNGDNRSESSKNKD 420

MN636841.1_LSDV090 ISKKFSTITGSYSESTKRIMFSHISHSISITDVSIPVNLWSCQRNVYNGDNRSESSKNKD 420

MN636842.1_LSDV090 ISKKFSTITGSYSESTKRIMFSHISHSISITDVSIPVNLWSCQRNVYNGDNRSESSKNKD 420

MN636843.1_LSDV090 ISKKFSTITGSYSESTKRIMFSHISHSISITDVSIPVNLWSCQRNVYNGDNRSESSKNKD 420

MT992618.1_LSDV090 ISKKFSTITGSYSESTKRIMFSHISHSISITDVSIPVNLWSCQRNVYNGDNRSESSKNKD 420

************************************************************

AF325528.1_LSDV090 LFINDPFIRGIDFKNKTDIISRMEVRFGNDVLYSETNPISKIYNDLLSNCVLGTRTLKFN 480

AF409137.1_LSDV090 LFINDPFIRGIDFKNKTDIISRMEVRFGNDVLYSETNPISKIYNDLLSNCVLGTRTLKFN 480

KX683219.1_LSDV090 LFINDPFIRGIDFKNKTDIISRMEVRFGNDVLYSETNPISKIYNDLLSNCVLGTRTLKFN 480

KX894508.1_LSDV090 LFINDPFIRGIDFKNKTDIISRMEVRFGNDVLYSETNPISKIYNDLLSNCVLGTRTLKFN 480

KY702007.1_LSDV090 LFINDPFIRGIDFKNKTDIISRMEVRFGNDVLYSETNPISKIYNDLLSNCVLGTRTLKFN 480

KY829023.3_LSDV090 LFINDPFIRGIDFKNKTDIISRMEVRFGNDVLYSETNPISKIYNDLLSNCVLGTRTLKFN 480

MH893760.2_LSDV090 LFINDPFIRGIDFKNKTDIISRMEVRFGNDVLYSETNPISKIYNDLLSNCVLGTRTLKFN 480

MN072619.1_LSDV090 LFINDPFIRGIDFKNKTDIISRMEVRFGNDVLYSETNPISKIYNDLLSNCVLGTRTLKFN 480

MN642592.1_LSDV090 LFINDPFIRGIDFKNKTDIISRMEVRFGNDVLYSETNPISKIYNDLLSNCVLGTRTLKFN 480

MN995838.1_LSDV090 LFINDPFIRGIDFKNKTDIISRMEVRFGNDVLYSETNPISKIYNDLLSNCVLGTRTLKFN 480

MT130502.2_LSDV090 LFINDPFIRGIDFKNKTDIISRMEVRFGNDVLYSETNPISKIYNDLLSNCVLGTRTLKFN 480

NC_003027.1_LSDV090 LFINDPFIRGIDFKNKTDIISRMEVRFGNDVLYSETNPISKIYNDLLSNCVLGTRTLKFN 480

AF409138.1_LSDV090 LFINDPFIRGIDFKNKTDIISRMEVRFGNDVLYSETNPISKIYNDLLSNCVLGTRTLKFN 480

HB977629.1_LSDV090 LFINDPFIRGIDFKNKTDIISRMEVRFGNDVLYSETNPISKIYNDLLSNCVLGTRTLKFN 480

KX764643.1_LSDV090 LFINDPFIRGIDFKNKTDIISRMEVRFGNDVLYSETNPISKIYNDLLSNCVLGTRTLKFN 480

KX764644.1_LSDV090 LFINDPFIRGIDFKNKTDIISRMEVRFGNDVLYSETNPISKIYNDLLSNCVLGTRTLKFN 480

KX764645.1_LSDV090 LFINDPFIRGIDFKNKTDIISRMEVRFGNDVLYSETNPISKIYNDLLSNCVLGTRTLKFN 480

MG972412.1_LSDV090 LFINDPFIRGIDFKNKTDIISRMEVRFGNDVLYSETNPISKIYNDLLSNCVLGTRTLKFN 480

MH646674.1_LSDV090 LFINDPFIRGIDFKNKTDIISRMEVRFGNDVLYSETNPISKIYNDLLSNCVLGTRTLKFN 480

MK441838.1_LSDV090 LFINDPFIRGIDFKNKTDIISRMEVRFGNDVLYSETNPISKIYNDLLSNCVLGTRTLKFN 480

MN636838.1_LSDV090 LFINDPFIRGIDFKNKTDIISRMEVRFGNDVLYSETNPISKIYNDLLSNCVLGTRTLKFN 480

MN636839.1_LSDV090 LFINDPFIRGIDFKNKTDIISRMEVRFGNDVLYSETNPISKIYNDLLSNCVLGTRTLKFN 480

MN636840.1_LSDV090 LFINDPFIRGIDFKNKTDIISRMEVRFGNDVLYSETNPISKIYNDLLSNCVLGTRTLKFN 480

MN636841.1_LSDV090 LFINDPFIRGIDFKNKTDIISRMEVRFGNDVLYSETNPISKIYNDLLSNCVLGTRTLKFN 480

MN636842.1_LSDV090 LFINDPFIRGIDFKNKTDIISRMEVRFGNDVLYSETNPISKIYNDLLSNCVLGTRTLKFN 480

MN636843.1_LSDV090 LFINDPFIRGIDFKNKTDIISRMEVRFGNDVLYSETNPISKIYNDLLSNCVLGTRTLKFN 480

MT992618.1_LSDV090 LFINDPFIRGIDFKNKTDIISRMEVRFGNDVLYSETNPISKIYNDLLSNCVLGTRTLKFN 480

************************************************************

AF325528.1_LSDV090 FTPHTFFKPTTIVSNTARGKDKLSVRVIFSSFDPNNPIYYVSKQIVLVCNDLYKVTNENG 540

AF409137.1_LSDV090 FTPHTFFKPTTIVSNTARGKDKLSVRVIFSSFDPNNPIYYVSKQIVLVCNDLYKVTNENG 540

KX683219.1_LSDV090 FTPHTFFKPTTIVSNTARGKDKLSVRVIFSSFDPNNPIYYVSKQIVLVCNDLYKVTNENG 540

KX894508.1_LSDV090 FTPHTFFKPTTIVSNTARGKDKLSVRVIFSSFDPNNPIYYVSKQIVLVCNDLYKVTNENG 540

KY702007.1_LSDV090 FTPHTFFKPTTIVSNTARGKDKLSVRVIFSSFDPNNPIYYVSKQIVLVCNDLYKVTNENG 540

KY829023.3_LSDV090 FTPHTFFKPTTIVSNTARGKDKLSVRVIFSSFDPNNPIYYVSKQIVLVCNDLYKVTNENG 540

MH893760.2_LSDV090 FTPHTFFKPTTIVSNTARGKDKLSVRVIFSSFDPNNPIYYVSKQIVLVCNDLYKVTNENG 540

MN072619.1_LSDV090 FTPHTFFKPTTIVSNTARGKDKLSVRVIFSSFDPNNPIYYVSKQIVLVCNDLYKVTNENG 540

MN642592.1_LSDV090 FTPHTFFKPTTIVSNTARGKDKLSVRVIFSSFDPNNPIYYVSKQIVLVCNDLYKVTNENG 540

MN995838.1_LSDV090 FTPHTFFKPTTIVSNTARGKDKLSVRVIFSSFDPNNPIYYVSKQIVLVCNDLYKVTNENG 540

MT130502.2_LSDV090 FTPHTFFKPTTIVSNTARGKDKLSVRVIFSSFDPNNPIYYVSKQIVLVCNDLYKVTNENG 540

NC_003027.1_LSDV090 FTPHTFFKPTTIVSNTARGKDKLSVRVIFSSFDPNNPIYYVSKQIVLVCNDLYKVTNENG 540

AF409138.1_LSDV090 FTPHTFFKPTTIVSNTARGKDKLSVRVIFSSFDPNNPIYYVSKQIVLVCNDLYKVTNENG 540

HB977629.1_LSDV090 FTPHTFFKPTTIVSNTARGKDKLSVRVIFSSFDPNNPIYYVSKQIVLVCNDLYKVTNENG 540

KX764643.1_LSDV090 FTPHTFFKPTTIVSNTARGKDKLSVRVIFSSFDPNNPIYYVSKQIVLVCNDLYKVTNENG 540

KX764644.1_LSDV090 FTPHTFFKPTTIVSNTARGKDKLSVRVIFSSFDPNNPIYYVSKQIVLVCNDLYKVTNENG 540

KX764645.1_LSDV090 FTPHTFFKPTTIVSNTARGKDKLSVRVIFSSFDPNNPIYYVSKQIVLVCNDLYKVTNENG 540

MG972412.1_LSDV090 FTPHTFFKPTTIVSNTARGKDKLSVRVIFSSFDPNNPIYYVSKQIVLVCNDLYKVTNENG 540

MH646674.1_LSDV090 FTPHTFFKPTTIVSNTARGKDKLSVRVIFSSFDPNNPIYYVSKQIVLVCNDLYKVTNENG 540

MK441838.1_LSDV090 FTPHTFFKPTTIVSNTARGKDKLSVRVIFSSFDPNNPIYYVSKQIVLVCNDLYKVTNENG 540

MN636838.1_LSDV090 FTPHTFFKPTTIVSNTARGKDKLSVRVIFSSFDPNNPIYYVSKQIVLVCNDLYKVTNENG 540

MN636839.1_LSDV090 FTPHTFFKPTTIVSNTARGKDKLSVRVIFSSFDPNNPIYYVSKQIVLVCNDLYKVTNENG 540

MN636840.1_LSDV090 FTPHTFFKPTTIVSNTARGKDKLSVRVIFSSFDPNNPIYYVSKQIVLVCNDLYKVTNENG 540

MN636841.1_LSDV090 FTPHTFFKPTTIVSNTARGKDKLSVRVIFSSFDPNNPIYYVSKQIVLVCNDLYKVTNENG 540

MN636842.1_LSDV090 FTPHTFFKPTTIVSNTARGKDKLSVRVIFSSFDPNNPIYYVSKQIVLVCNDLYKVTNENG 540

MN636843.1_LSDV090 FTPHTFFKPTTIVSNTARGKDKLSVRVIFSSFDPNNPIYYVSKQIVLVCNDLYKVTNENG 540

MT992618.1_LSDV090 FTPHTFFKPTTIVSNTARGKDKLSVRVIFSSFDPNNPIYYVSKQIVLVCNDLYKVTNENG 540

************************************************************

AF325528.1_LSDV090 INVIKITEE 549

AF409137.1_LSDV090 INVIKITEE 549

KX683219.1_LSDV090 INVIKITEE 549

KX894508.1_LSDV090 INVIKITEE 549

KY702007.1_LSDV090 INVIKITEE 549

KY829023.3_LSDV090 INVIKITEE 549

MH893760.2_LSDV090 INVIKITEE 549

MN072619.1_LSDV090 INVIKITEE 549

MN642592.1_LSDV090 INVIKITEE 549

MN995838.1_LSDV090 INVIKITEE 549

MT130502.2_LSDV090 INVIKITEE 549

NC_003027.1_LSDV090 INVIKITEE 549

AF409138.1_LSDV090 INVIKITEE 549

HB977629.1_LSDV090 INVIKITEE 549

KX764643.1_LSDV090 INVIKITEE 549

KX764644.1_LSDV090 INVIKITEE 549

KX764645.1_LSDV090 INVIKITEE 549

MG972412.1_LSDV090 INVIKITEE 549

MH646674.1_LSDV090 INVIKITEE 549

MK441838.1_LSDV090 INVIKITEE 549

MN636838.1_LSDV090 INVIKITEE 549

MN636839.1_LSDV090 INVIKITEE 549

MN636840.1_LSDV090 INVIKITEE 549

MN636841.1_LSDV090 INVIKITEE 549

MN636842.1_LSDV090 INVIKITEE 549

MN636843.1_LSDV090 INVIKITEE 549

MT992618.1_LSDV090 INVIKITEE 549

*********

**Multiple alignment of LSDV0109 from 27 isolates of LSDV**

AF325528.1_LSDV109 MSYLSYYNMFSDFTAGAGVSDNELFTREEEEAFLPKEHHEEGEEYKISKHHSLKNRFPNI 60

AF409137.1_LSDV109 MSYLSYYNMFSDFTAGAGVSDNELFTREEEEAFLPKEHHEEGEEYKISKHHSLKNRFPNI 60

KX683219.1_LSDV109 MSYLSYYNMFSDFTAGAGVSDNELFTREEEEAFLPKEHHEEGEEYKISKHHSLKNRFPNI 60

KX894508.1_LSDV109 MSYLSYYNMFSDFTAGAGVSDNELFTREEEEAFLPKEHHEEGEEYKISKHHSLKNRFPNI 60

KY702007.1_LSDV109 MSYLSYYNMFSDFTAGAGVSDNELFTREEEEAFLPKEHHEEGEEYKISKHHSLKNRFPNI 60

KY829023.3_LSDV109 MSYLSYYNMFSDFTAGAGVSDNELFTREEEEAFLPKEHHEEGEEYKISKHHSLKNRFPNI 60

MH893760.2_LSDV109 MSYLSYYNMFSDFTAGAGVSDNELFTREEEEAFLPKEHHEEGEEYKISKHHSLKNRFPNI 60

MN072619.1_LSDV109 MSYLSYYNMFSDFTAGAGVSDNELFTREEEEAFLPKEHHEEGEEYKISKHHSLKNRFPNI 60

MN642592.1_LSDV109 MSYLSYYNMFSDFTAGAGVSDNELFTREEEEAFLPKEHHEEGEEYKISKHHSLKNRFPNI 60

MN995838.1_LSDV109 MSYLSYYNMFSDFTAGAGVSDNELFTREEEEAFLPKEHHEEGEEYKISKHHSLKNRFPNI 60

MT130502.2_LSDV109 MSYLSYYNMFSDFTAGAGVSDNELFTREEEEAFLPKEHHEEGEEYKISKHHSLKNRFPNI 60

NC_003027.1_LSDV109 MSYLSYYNMFSDFTAGAGVSDNELFTREEEEAFLPKEHHEEGEEYKISKHHSLKNRFPNI 60

AF409138.1_LSDV109 MSYLSYYNMFSDFTAGAGVSDNELFTREEEEAFLPKEHHEEGEEYKISKHHSLKNRFPNI 60

HB977629.1_LSDV109 MSYLSYYNMFSDFTAGAGVSDNELFTREEEEAFLPKEHHEEGEEYKISKHHSLKNRFPNI 60

KX764643.1_LSDV109 MSYLSYYNMFSDFTAGAGVSDNELFTREEEEAFLPKEHHEEGEEYKISKHHSLKNRFPNI 60

KX764644.1_LSDV109 MSYLSYYNMFSDFTAGAGVSDNELFTREEEEAFLPKEHHEEGEEYKISKHHSLKNRFPNI 60

KX764645.1_LSDV109 MSYLSYYNMFSDFTAGAGVSDNELFTREEEEAFLPKEHHEEGEEYKISKHHSLKNRFPNI 60

MG972412.1_LSDV109 MSYLSYYNMFSDFTAGAGVSDNELFTREEEEAFLPKEHHEEGEEYKISKHHSLKNRFPNI 60

MH646674.1_LSDV109 MSYLSYYNMFSDFTAGAGVSDNELFTREEEEAFLPKEHHEEGEEYKISKHHSLKNRFPNI 60

MK441838.1_LSDV109 MSYLSYYNMFSDFTAGAGVSDNELFTREEEEAFLPKEHHEEGEEYKISKHHSLKNRFPNI 60

MN636838.1_LSDV109 MSYLSYYNMFSDFTAGAGVSDNELFTREEEEAFLPKEHHEEGEEYKISKHHSLKNRFPNI 60

MN636839.1_LSDV109 MSYLSYYNMFSDFTAGAGVSDNELFTREEEEAFLPKEHHEEGEEYKISKHHSLKNRFPNI 60

MN636840.1_LSDV109 MSYLSYYNMFSDFTAGAGVSDNELFTREEEEAFLPKEHHEEGEEYKISKHHSLKNRFPNI 60

MN636841.1_LSDV109 MSYLSYYNMFSDFTAGAGVSDNELFTREEEEAFLPKEHHEEGEEYKISKHHSLKNRFPNI 60

MN636842.1_LSDV109 MSYLSYYNMFSDFTAGAGVSDNELFTREEEEAFLPKEHHEEGEEYKISKHHSLKNRFPNI 60

MN636843.1_LSDV109 MSYLSYYNMFSDFTAGAGVSDNELFTREEEEAFLPKEHHEEGEEYKISKHHSLKNRFPNI 60

MT992618.1_LSDV109 MSYLSYYNMFSDFTAGAGVSDNELFTREEEEAFLPKEHHEEGEEYKISKHHSLKNRFPNI 60

************************************************************

AF325528.1_LSDV109 LMRSDIRALIGLILFVLAITTTPIIAVIMIAVASALLPFPSLVIAYCLSIQKFSGGVGNN 120

AF409137.1_LSDV109 LMRSDIRALIGLILFVLAITTTPIIAVIMIAVASALLPFPSLVIAYCLSIQKFSGGVGNN 120

KX683219.1_LSDV109 LMRSDIRALIGLILFVLAITTTPIIAVIMIAVASALLPFPSLVIAYCLSIQKFSGGVGNN 120

KX894508.1_LSDV109 LMRSDIRALIGLILFVLAITTTPIIAVIMIAVASALLPFPSLVIAYCLSIQKFSGGVGNN 120

KY702007.1_LSDV109 LMRSDIRALIGLILFVLAITTTPIIAVIMIAVASALLPFPSLVIAYCLSIQKFSGGVGNN 120

KY829023.3_LSDV109 LMRSDIRALIGLILFVLAITTTPIIAVIMIAVASALLPFPSLVIAYCLSIQKFSGGVGNN 120

MH893760.2_LSDV109 LMRSDIRALIGLILFVLAITTTPIIAVIMIAVASALLPFPSLVIAYCLSIQKFSGGVGNN 120

MN072619.1_LSDV109 LMRSDIRALIGLILFVLAITTTPIIAVIMIAVASALLPFPSLVIAYCLSIQKFSGGVGNN 120

MN642592.1_LSDV109 LMRSDIRALIGLILFVLAITTTPIIAVIMIAVASALLPFPSLVIAYCLSIQKFSGGVGNN 120

MN995838.1_LSDV109 LMRSDIRALIGLILFVLAITTTPIIAVIMIAVASALLPFPSLVIAYCLSIQKFSGGVGNN 120

MT130502.2_LSDV109 LMRSDIRALIGLILFVLAITTTPIIAVIMIAVASALLPFPSLVIAYCLSIQKFSGGVGNN 120

NC_003027.1_LSDV109 LMRSDIRALIGLILFVLAITTTPIIAVIMIAVASALLPFPSLVIAYCLSIQKFSGGVGNN 120

AF409138.1_LSDV109 LMRSDIRALIGLILFVLAITTTPIIAVIMIAVASALLPFPSLVIAYCLSIQKFSGGVGNN 120

HB977629.1_LSDV109 LMRSDIRALIGLILFVLAITTTPIIAVIMIAVASALLPFPSLVIAYCLSIQKFSGGVGNN 120

KX764643.1_LSDV109 LMRSDIRALIGLILFVLAITTTPIIAVIMIAVASALLPFPSLVIAYCLSIQKFSGGVGNN 120

KX764644.1_LSDV109 LMRSDIRALIGLILFVLAITTTPIIAVIMIAVASALLPFPSLVIAYCLSIQKFSGGVGNN 120

KX764645.1_LSDV109 LMRSDIRALIGLILFVLAITTTPIIAVIMIAVASALLPFPSLVIAYCLSIQKFSGGVGNN 120

MG972412.1_LSDV109 LMRSDIRALIGLILFVLAITTTPIIAVIMIAVASALLPFPSLVIAYCLSIQKFSGGVGNN 120

MH646674.1_LSDV109 LMRSDIRALIGLILFVLAITTTPIIAVIMIAVASALLPFPSLVIAYCLSIQKFSGGVGNN 120

MK441838.1_LSDV109 LMRSDIRALIGLILFVLAITTTPIIAVIMIAVASALLPFPSLVIAYCLSIQKFSGGVGNN 120

MN636838.1_LSDV109 LMRSDIRALIGLILFVLAITTTPIIAVIMIAVASALLPFPSLVIAYCLSIQKFSGGVGNN 120

MN636839.1_LSDV109 LMRSDIRALIGLILFVLAITTTPIIAVIMIAVASALLPFPSLVIAYCLSIQKFSGGVGNN 120

MN636840.1_LSDV109 LMRSDIRALIGLILFVLAITTTPIIAVIMIAVASALLPFPSLVIAYCLSIQKFSGGVGNN 120

MN636841.1_LSDV109 LMRSDIRALIGLILFVLAITTTPIIAVIMIAVASALLPFPSLVIAYCLSIQKFSGGVGNN 120

MN636842.1_LSDV109 LMRSDIRALIGLILFVLAITTTPIIAVIMIAVASALLPFPSLVIAYCLSIQKFSGGVGNN 120

MN636843.1_LSDV109 LMRSDIRALIGLILFVLAITTTPIIAVIMIAVASALLPFPSLVIAYCLSIQKFSGGVGNN 120

MT992618.1_LSDV109 LMRSDIRALIGLILFVLAITTTPIIAVIMIAVASALLPFPSLVIAYCLSIQKFSGGVGNN 120

************************************************************

AF325528.1_LSDV109 KQIIMSILCVSVSIITLIFRHISSTAYTISYIILAILFCVYAFNISKISYQDQDQACYKN 180

AF409137.1_LSDV109 KQIIMSILCVSVSIITLIFRHISSTAYTISYIILAILFCVYAFNISKISYQDQDQACYKN 180

KX683219.1_LSDV109 KQIIMSILCVSVSIITLIFRHISSTAYTISYIILAILFCVYAFNISKISYQDQDQACYKN 180

KX894508.1_LSDV109 KQIIMSILCVSVSIITLIFRHISSTAYTISYIILAILFCVYAFNISKISYQDQDQACYKN 180

KY702007.1_LSDV109 KQIIMSILCVSVSIITLIFRHISSTAYTISYIILAILFCVYAFNISKISYQDQDQACYKN 180

KY829023.3_LSDV109 KQIIMSILCVSVSIITLIFRHISSTAYTISYIILAILFCVYAFNISKISYQDQDQACYKN 180

MH893760.2_LSDV109 KQIIMSILCVSVSIITLIFRHISSTAYTISYIILAILFCVYAFNISKISYQDQDQACYKN 180

MN072619.1_LSDV109 KQIIMSILCVSVSIITLIFRHISSTAYTISYIILAILFCVYAFNISKISYQDQDQACYKN 180

MN642592.1_LSDV109 KQIIMSILCVSVSIITLIFRHISSTAYTISYIILAILFCVYAFNISKISYQDQDQACYKN 180

MN995838.1_LSDV109 KQIIMSILCVSVSIITLIFRHISSTAYTISYIILAILFCVYAFNISKISYQDQDQACYKN 180

MT130502.2_LSDV109 KQIIMSILCVSVSIITLIFRHISSTAYTISYIILAILFCVYAFNISKISYQDQDQACYKN 180

NC_003027.1_LSDV109 KQIIMSILCVSVSIITLIFRHISSTAYTISYIILAILFCVYAFNISKISYQDQDQACYKN 180

AF409138.1_LSDV109 KQIIMSILCVSVSIITLIFRHISSTAYTISYIILAILFCVYAFNISKISYQDQDQACYKN 180

HB977629.1_LSDV109 KQIIMSILCVSVSIITLIFRHISSTAYTISYIILAILFCVYAFNISKISYQDQDQACYKN 180

KX764643.1_LSDV109 KQIIMSILCVSVSIITLIFRHISSTAYTISYIILAILFCVYAFNISKISYQDQDQACYKN 180

KX764644.1_LSDV109 KQIIMSILCVSVSIITLIFRHISSTAYTISYIILAILFCVYAFNISKISYQDQDQACYKN 180

KX764645.1_LSDV109 KQIIMSILCVSVSIITLIFRHISSTAYTISYIILAILFCVYAFNISKISYQDQDQACYKN 180

MG972412.1_LSDV109 KQIIMSILCVSVSIITLIFRHISSTAYTISYIILAILFCVYAFNISKISYQDQDQACYKN 180

MH646674.1_LSDV109 KQIIMSILCVSVSIITLIFRHISSTAYTISYIILAILFCVYAFNISKISYQDQDQACYKN 180

MK441838.1_LSDV109 KQIIMSILCVSVSIITLIFRHISSTAYTISYIILAILFCVYAFNISKISYQDQDQACYKN 180

MN636838.1_LSDV109 KQIIMSILCVSVSIITLIFRHISSTAYTISYIILAILFCVYAFNISKISYQDQDQACYKN 180

MN636839.1_LSDV109 KQIIMSILCVSVSIITLIFRHISSTAYTISYIILAILFCVYAFNISKISYQDQDQACYKN 180

MN636840.1_LSDV109 KQIIMSILCVSVSIITLIFRHISSTAYTISYIILAILFCVYAFNISKISYQDQDQACYKN 180

MN636841.1_LSDV109 KQIIMSILCVSVSIITLIFRHISSTAYTISYIILAILFCVYAFNISKISYQDQDQACYKN 180

MN636842.1_LSDV109 KQIIMSILCVSVSIITLIFRHISSTAYTISYIILAILFCVYAFNISKISYQDQDQACYKN 180

MN636843.1_LSDV109 KQIIMSILCVSVSIITLIFRHISSTAYTISYIILAILFCVYAFNISKISYQDQDQACYKN 180

MT992618.1_LSDV109 KQIIMSILCVSVSIITLIFRHISSTAYTISYIILAILFCVYAFNISKISYQDQDQACYKN 180

************************************************************

AF325528.1_LSDV109 FKGGNKYREKPSFYEE 196

AF409137.1_LSDV109 FKGGNKYREKPSFYEE 196

KX683219.1_LSDV109 FKGGNKYREKPSFYEE 196

KX894508.1_LSDV109 FKGGNKYREKPSFYEE 196

KY702007.1_LSDV109 FKGGNKYREKPSFYEE 196

KY829023.3_LSDV109 FKGGNKYREKPSFYEE 196

MH893760.2_LSDV109 FKGGNKYREKPSFYEE 196

MN072619.1_LSDV109 FKGGNKYREKPSFYEE 196

MN642592.1_LSDV109 FKGGNKYREKPSFYEE 196

MN995838.1_LSDV109 FKGGNKYREKPSFYEE 196

MT130502.2_LSDV109 FKGGNKYREKPSFYEE 196

NC_003027.1_LSDV109 FKGGNKYREKPSFYEE 196

AF409138.1_LSDV109 vKGGNKYREKPSFYEE 196

HB977629.1_LSDV109 vKGGNKYREKPSFYEE 196

KX764643.1_LSDV109 vKGGNKYREKPSFYEE 196

KX764644.1_LSDV109 vKGGNKYREKPSFYEE 196

KX764645.1_LSDV109 vKGGNKYREKPSFYEE 196

MG972412.1_LSDV109 vKGGNKYREKPSFYEE 196

MH646674.1_LSDV109 vKGGNKYREKPSFYEE 196

MK441838.1_LSDV109 vKGGNKYREKPSFYEE 196

MN636838.1_LSDV109 vKGGNKYREKPSFYEE 196

MN636839.1_LSDV109 vKGGNKYREKPSFYEE 196

MN636840.1_LSDV109 vKGGNKYREKPSFYEE 196

MN636841.1_LSDV109 vKGGNKYREKPSFYEE 196

MN636842.1_LSDV109 vKGGNKYREKPSFYEE 196

MN636843.1_LSDV109 vKGGNKYREKPSFYEE 196

MT992618.1_LSDV109 vKGGNKYREKPSFYEE 196

.***************

**Multiple alignment of LSDV103 from 27 isolates of LSDV**

AF325528.1_LSDV103 MSDKKLSRSSYDDYIETINKLTPQLRTILAHISGEQASQKSNLTPEDNTTNNTDENEVKA 60

HB977629.1_LSDV103 MSDKKLSRSSYDDYIETINKLTPQLRTILAHISGEQASQKSNLTPEDNTTNNTDENEVKA 60

KX683219.1_LSDV103 MSDKKLSRSSYDDYIETINKLTPQLRTILAHISGEQASQKSNLTPEDNTTNNTDENEVKA 60

MH646674.1_LSDV103 MSDKKLSRSSYDDYIETINKLTPQLRTILAHISGEQASQKSNLTPEDNTTNNTDENEVKA 60

MN072619.1_LSDV103 MSDKKLSRSSYDDYIETINKLTPQLRTILAHISGEQASQKSNLTPEDNTTNNTDENEVKA 60

MN995838.1_LSDV103 MSDKKLSRSSYDDYIETINKLTPQLRTILAHISGEQASQKSNLTPEDNTTNNTDENEVKA 60

MT992618.1_LSDV103 MSDKKLSRSSYDDYIETINKLTPQLRTILAHISGEQASQKSNLTPEDNTTNNTDENEVKA 60

NC_003027.1_LSDV103 MSDKKLSRSSYDDYIETINKLTPQLRTILAHISGEQASQKSNLTPEDNTTNNTDENEVKA 60

AF409137.1_LSDV103 MSDKKLSRSSYDDYIETINKLTPQLRTILAHISGEQASQKSNLTPEDNTTNNTDENEVKA 60

KX894508.1_LSDV103 MSDKKLSRSSYDDYIETINKLTPQLRTILAHISGEQASQKSNLTPEDNTTNNTDENEVKA 60

KY702007.1_LSDV103 MSDKKLSRSSYDDYIETINKLTPQLRTILAHISGEQASQKSNLTPEDNTTNNTDENEVKA 60

KY829023.3_LSDV103 MSDKKLSRSSYDDYIETINKLTPQLRTILAHISGEQASQKSNLTPEDNTTNNTDENEVKA 60

MH893760.2_LSDV103 MSDKKLSRSSYDDYIETINKLTPQLRTILAHISGEQASQKSNLTPEDNTTNNTDENEVKA 60

MN642592.1_LSDV103 MSDKKLSRSSYDDYIETINKLTPQLRTILAHISGEQASQKSNLTPEDNTTNNTDENEVKA 60

MT130502.2_LSDV103 MSDKKLSRSSYDDYIETINKLTPQLRTILAHISGEQASQKSNLTPEDNTTNNTDENEVKA 60

MN636841.1_LSDV103 MSDKKLSRSSYDDYIETINKLTPQLRTILAHISGEQASQKSNLTPEDNTnNNTDENEVKA 60

KX764643.1_LSDV103 MSDKKLSRSSYDDYIETINKLTPQLRTILAHISGEQASQKSNLTPEDNTnNNTDENEVKA 60

AF409138.1_LSDV103 MSDKKLSRSSYDDYIETINKLTPQLRTILAHISGEQASQKSNLTPEDNTnNNTDENEVKA 60

KX764644.1_LSDV103 MSDKKLSRSSYDDYIETINKLTPQLRTILAHISGEQASQKSNLTPEDNTnNNTDENEVKA 60

KX764645.1_LSDV103 MSDKKLSRSSYDDYIETINKLTPQLRTILAHISGEQASQKSNLTPEDNTnNNTDENEVKA 60

MG972412.1_LSDV103 MSDKKLSRSSYDDYIETINKLTPQLRTILAHISGEQASQKSNLTPEDNTnNNTDENEVKA 60

MK441838.1_LSDV103 MSDKKLSRSSYDDYIETINKLTPQLRTILAHISGEQASQKSNLTPEDNTnNNTDENEVKA 60

MN636838.1_LSDV103 MSDKKLSRSSYDDYIETINKLTPQLRTILAHISGEQASQKSNLTPEDNTnNNTDENEVKA 60

MN636839.1_LSDV103 MSDKKLSRSSYDDYIETINKLTPQLRTILAHISGEQASQKSNLTPEDNTnNNTDENEVKA 60

MN636840.1_LSDV103 MSDKKLSRSSYDDYIETINKLTPQLRTILAHISGEQASQKSNLTPEDNTnNNTDENEVKA 60

MN636842.1_LSDV103 MSDKKLSRSSYDDYIETINKLTPQLRTILAHISGEQASQKSNLTPEDNTnNNTDENEVKA 60

MN636843.1_LSDV103 MSDKKLSRSSYDDYIETINKLTPQLRTILAHISGEQASQKSNLTPEDNTnNNTDENEVKA 60

*************************************************.**********

AF325528.1_LSDV103 GNVKTKACMTKPNKKSKSCSNKQTTSRSSNVCSSKSVNNGAVFKKRNTFNETDQIMQAVT 120

HB977629.1_LSDV103 GNVKTKACMTKPNKKSKSCSNKQTTSRSSNVCSSKSVNNGAVFKKRNTFNETDQIMQAVT 120

KX683219.1_LSDV103 GNVKTKACMTKPNKKSKSCSNKQTTSRSSNVCSSKSVNNGAVFKKRNTFNETDQIMQAVT 120

MH646674.1_LSDV103 GNVKTKACMTKPNKKSKSCSNKQTTSRSSNVCSSKSVNNGAVFKKRNTFNETDQIMQAVT 120

MN072619.1_LSDV103 GNVKTKACMTKPNKKSKSCSNKQTTSRSSNVCSSKSVNNGAVFKKRNTFNETDQIMQAVT 120

MN995838.1_LSDV103 GNVKTKACMTKPNKKSKSCSNKQTTSRSSNVCSSKSVNNGAVFKKRNTFNETDQIMQAVT 120

MT992618.1_LSDV103 GNVKTKACMTKPNKKSKSCSNKQTTSRSSNVCSSKSVNNGAVFKKRNTFNETDQIMQAVT 120

NC_003027.1_LSDV103 GNVKTKACMTKPNKKSKSCSNKQTTSRSSNVCSSKSVNNGAVFKKRNTFNETDQIMQAVT 120

AF409137.1_LSDV103 GNVKTKACMTKPNKKSKSCSNKQTTSRSgNVCSSKSVNNGAVFKKRNTFNETDQIMQAVT 120

KX894508.1_LSDV103 GNVKTKACMTKPNKKSKSCSNKQTTSRSgNVCSSKSVNNGAVFKKRNTFNETDQIMQAVT 120

KY702007.1_LSDV103 GNVKTKACMTKPNKKSKSCSNKQTTSRSgNVCSSKSVNNGAVFKKRNTFNETDQIMQAVT 120

KY829023.3_LSDV103 GNVKTKACMTKPNKKSKSCSNKQTTSRSgNVCSSKSVNNGAVFKKRNTFNETDQIMQAVT 120

MH893760.2_LSDV103 GNVKTKACMTKPNKKSKSCSNKQTTSRSgNVCSSKSVNNGAVFKKRNTFNETDQIMQAVT 120

MN642592.1_LSDV103 GNVKTKACMTKPNKKSKSCSNKQTTSRSgNVCSSKSVNNGAVFKKRNTFNETDQIMQAVT 120

MT130502.2_LSDV103 GNVKTKACMTKPNKKSKSCSNKQTTSRSgNVCSSKSVNNGAVFKKRNTFNETDQIMQAVT 120

MN636841.1_LSDV103 GNVKTKACMTKtNKKSKSCSNKQTTSRSSNVCSSKSVNNGAVFKKRNTFNETDQIMQAVT 120

KX764643.1_LSDV103 GNVKTKACMTtPNKKSKSCSNKQTTSRSgNVCSSKSVNNGAVFKKRNTFNETDQIMQAVT 120

AF409138.1_LSDV103 GNVKTKACMTKtNKKSKSCSNKQTTSRSgNVCSSKSVNNGAVFKKRNTFNETDQIMQAVT 120

KX764644.1_LSDV103 GNVKTKACMTKtNKKSKSCSNKQTTSRSgNVCSSKSVNNGAVFKKRNTFNETDQIMQAVT 120

KX764645.1_LSDV103 GNVKTKACMTKtNKKSKSCSNKQTTSRSgNVCSSKSVNNGAVFKKRNTFNETDQIMQAVT 120

MG972412.1_LSDV103 GNVKTKACMTKtNKKSKSCSNKQTTSRSgNVCSSKSVNNGAVFKKRNTFNETDQIMQAVT 120

MK441838.1_LSDV103 GNVKTKACMTKtNKKSKSCSNKQTTSRSgNVCSSKSVNNGAVFKKRNTFNETDQIMQAVT 120

MN636838.1_LSDV103 GNVKTKACMTKtNKKSKSCSNKQTTSRSgNVCSSKSVNNGAVFKKRNTFNETDQIMQAVT 120

MN636839.1_LSDV103 GNVKTKACMTKPNKKSKSCSNKQTTSRSgNVCSSKSVNNGAVFKKRNTFNETDQIMQAVT 120

MN636840.1_LSDV103 GNVKTKACMTKPNKKSKSCSNKQTTSRSgNVCSSKSVNNGAVFKKRNTFNETDQIMQAVT 120

MN636842.1_LSDV103 GNVKTKACMTKPNKKSKSCSNKQTTSRSgNVCSSKSVNNGAVFKKRNTFNETDQIMQAVT 120

MN636843.1_LSDV103 GNVKTKACMTKPNKKSKSCSNKQTTSRSgNVCSSKSVNNGAVFKKRNTFNETDQIMQAVT 120

**********. ****************.*******************************

AF325528.1_LSDV103 NGGKIVYGTMKEGKLEVQGMVGEINQDLLGIESVNAGRRNKNISQSKKKLIKRGMYKVET 180

HB977629.1_LSDV103 NGGKIVYGTMKEGKLEVQGMVGEINQDLLGIESVNAGRRNKNISQSKKKLIKRGMYKVET 180

KX683219.1_LSDV103 NGGKIVYGTMKEGKLEVQGMVGEINQDLLGIESVNAGRRNKNISQSKKKLIKRGMYKVET 180

MH646674.1_LSDV103 NGGKIVYGTMKEGKLEVQGMVGEINQDLLGIESVNAGRRNKNISQSKKKLIKRGMYKVET 180

MN072619.1_LSDV103 NGGKIVYGTMKEGKLEVQGMVGEINQDLLGIESVNAGRRNKNISQSKKKLIKRGMYKVET 180

MN995838.1_LSDV103 NGGKIVYGTMKEGKLEVQGMVGEINQDLLGIESVNAGRRNKNISQSKKKLIKRGMYKVET 180

MT992618.1_LSDV103 NGGKIVYGTMKEGKLEVQGMVGEINQDLLGIESVNAGRRNKNISQSKKKLIKRGMYKVET 180

NC_003027.1_LSDV103 NGGKIVYGTMKEGKLEVQGMVGEINQDLLGIESVNAGRRNKNISQSKKKLIKRGMYKVET 180

AF409137.1_LSDV103 NGGKIVYGTMKEGKLEVQGMVGEINQDLLGIESVNAGRRNKNISQSKKKLIKRGMYKVET 180

KX894508.1_LSDV103 NGGKIVYGTMKEGKLEVQGMVGEINQDLLGIESVNAGRRNKNISQSKKKLIKRGMYKVET 180

KY702007.1_LSDV103 NGGKIVYGTMKEGKLEVQGMVGEINQDLLGIESVNAGRRNKNISQSKKKLIKRGMYKVET 180

KY829023.3_LSDV103 NGGKIVYGTMKEGKLEVQGMVGEINQDLLGIESVNAGRRNKNISQSKKKLIKRGMYKVET 180

MH893760.2_LSDV103 NGGKIVYGTMKEGKLEVQGMVGEINQDLLGIESVNAGRRNKNISQSKKKLIKRGMYKVET 180

MN642592.1_LSDV103 NGGKIVYGTMKEGKLEVQGMVGEINQDLLGIESVNAGRRNKNISQSKKKLIKRGMYKVET 180

MT130502.2_LSDV103 NGGKIVYGTMKEGKLEVQGMVGEINQDLLGIESVNAGRRNKNISQSKKKLIKRGMYKVET 180

MN636841.1_LSDV103 NGGKIVYGTMKEGKLEVQGMVGEINQDLLGIESVNAGRRNKNISQSKKKLIKRGMYKVET 180

KX764643.1_LSDV103 NGGKIVYGTMKEGKLEVQGMVGEINQDLLGIESVNAGRRNKNISQSKKKLIKRGMYKVET 180

AF409138.1_LSDV103 NGGKIVYGTMKEGKLEVQGMVGEINQDLLGIESVNAGRRNKNISQSKKKLIKRGMYKVET 180

KX764644.1_LSDV103 NGGKIVYGTMKEGKLEVQGMVGEINQDLLGIESVNAGRRNKNISQSKKKLIKRGMYKVET 180

KX764645.1_LSDV103 NGGKIVYGTMKEGKLEVQGMVGEINQDLLGIESVNAGRRNKNISQSKKKLIKRGMYKVET 180

MG972412.1_LSDV103 NGGKIVYGTMKEGKLEVQGMVGEINQDLLGIESVNAGRRNKNISQSKKKLIKRGMYKVET 180

MK441838.1_LSDV103 NGGKIVYGTMKEGKLEVQGMVGEINQDLLGIESVNAGRRNKNISQSKKKLIKRGMYKVET 180

MN636838.1_LSDV103 NGGKIVYGTMKEGKLEVQGMVGEINQDLLGIESVNAGRRNKNISQSKKKLIKRGMYKVET 180

MN636839.1_LSDV103 NGGKIVYGTMKEGKLEVQGMVGEINQDLLGIESVNAGRRNKNISQSKKKLIKRGMYKVET 180

MN636840.1_LSDV103 NGGKIVYGTMKEGKLEVQGMVGEINQDLLGIESVNAGRRNKNISQSKKKLIKRGMYKVET 180

MN636842.1_LSDV103 NGGKIVYGTMKEGKLEVQGMVGEINQDLLGIESVNAGRRNKNISQSKKKLIKRGMYKVET 180

MN636843.1_LSDV103 NGGKIVYGTMKEGKLEVQGMVGEINQDLLGIESVNAGRRNKNISQSKKKLIKRGMYKVET 180

************************************************************

AF325528.1_LSDV103 ADDSIDDGMD 190

HB977629.1_LSDV103 ADDSIDDGMD 190

KX683219.1_LSDV103 ADDSIDDGMD 190

MH646674.1_LSDV103 ADDSIDDGMD 190

MN072619.1_LSDV103 ADDSIDDGMD 190

MN995838.1_LSDV103 ADDSIDDGMD 190

MT992618.1_LSDV103 ADDSIDDGMD 190

NC_003027.1_LSDV103 ADDSIDDGMD 190

AF409137.1_LSDV103 ADDSIDDGMD 190

KX894508.1_LSDV103 ADDSIDDGMD 190

KY702007.1_LSDV103 ADDSIDDGMD 190

KY829023.3_LSDV103 ADDSIDDGMD 190

MH893760.2_LSDV103 ADDSIDDGMD 190

MN642592.1_LSDV103 ADDSIDDGMD 190

MT130502.2_LSDV103 ADDSIDDGMD 190

MN636841.1_LSDV103 ADDSIDDGMD 190

KX764643.1_LSDV103 ADDSIDDGMD 190

AF409138.1_LSDV103 ADDSIDDGMD 190

KX764644.1_LSDV103 ADDSIDDGMD 190

KX764645.1_LSDV103 ADDSIDDGMD 190

MG972412.1_LSDV103 ADDSIDDGMD 190

MK441838.1_LSDV103 ADDSIDDGMD 190

MN636838.1_LSDV103 ADDSIDDGMD 190

MN636839.1_LSDV103 ADDSIDDGMD 190

MN636840.1_LSDV103 ADDSIDDGMD 190

MN636842.1_LSDV103 ADDSIDDGMD 190

MN636843.1_LSDV103 ADDSIDDGMD 190

**********
